# Supplementary material for: Rubicon modulates neuroimmune responses following traumatic brain injury
Source: Cell Death Dis. 2026 Jul 27;17(1):899. doi: 10.1038/s41419-026-09083-4 (PMC13687110; doi:10.1038/s41419-026-09083-4)
Supplement: Supplementary file 1 [file 41419_2026_9083_MOESM1_ESM.pdf]

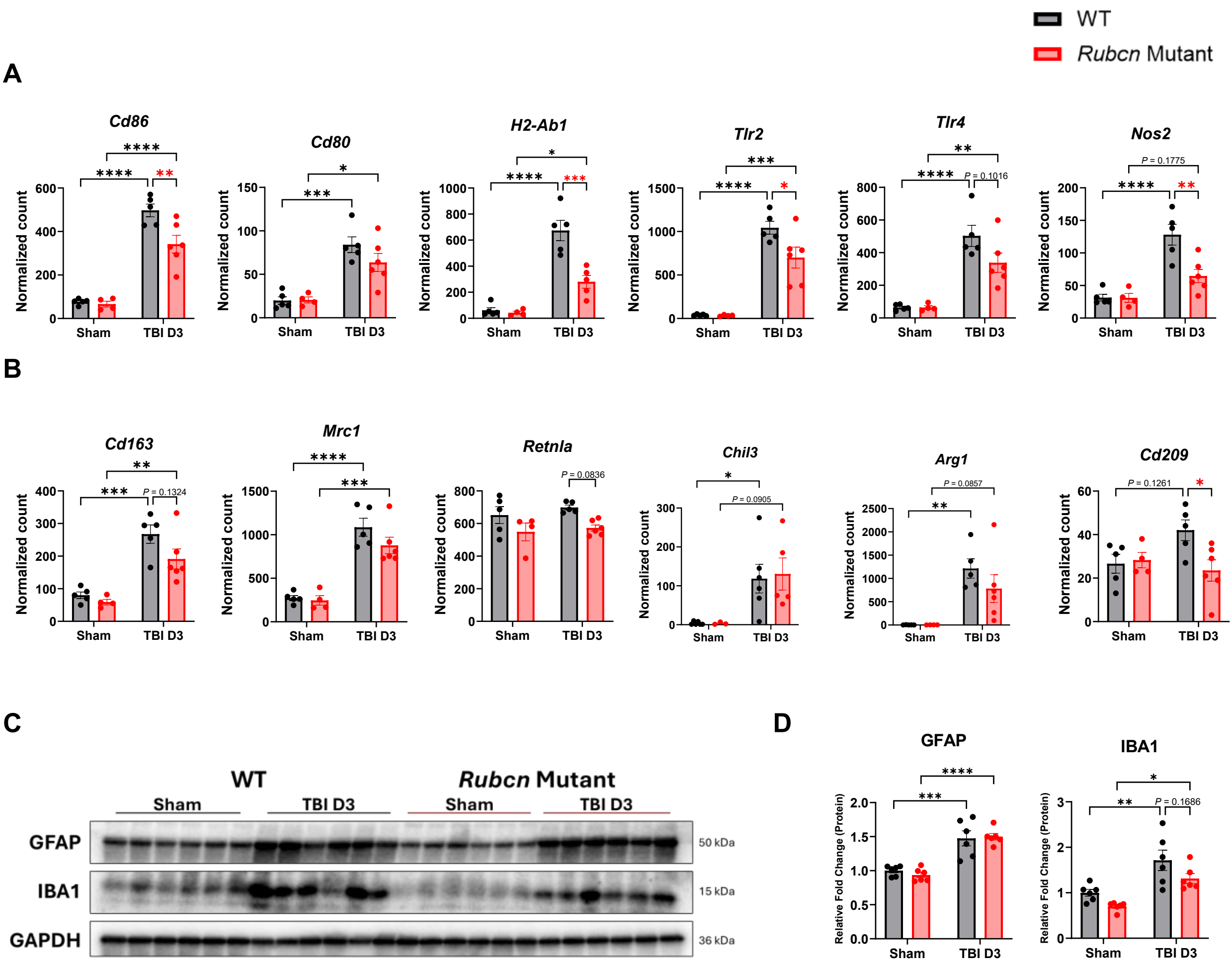

Supplemental Figure 1: Rubicon mutation decreases microglial pro-inflammatory polarization. **A** Gene expression of pro-inflammatory M1-like genes in the ipsilateral cortices of wild-type and *Rubcn*-mutant mice at 3 dpi (TBI D3) in comparison to sham mice. **B** Gene expression of anti-inflammatory M2-like genes in the ipsilateral cortices of wild-type and *Rubcn*-mutant mice at 3 dpi (TBI D3) in comparison to sham. Levels of gene expression are based on RNA-seq analysis and represented as normalized counts for **A** & **B**. **C** Immunoblot comparing GFAP and IBA1 expression in the ipsilateral cortices from wild-type and *Rubcn*-mutant mice at 3 dpi to sham. **D** Densitometric quantification of immunoblot in **C**. Bars represent mean ± s.e.m. Sample size (*n*) for **A-B** were as follows: WT Sham=5, WT TBI D3=5, *Rubcn* Mut. Sham=4, *Rubcn* Mut. TBI D3=6. Sample size (*n*) for **C-D** were as follows: WT Sham=6, WT TBI D3=6, *Rubcn* Mut. Sham=6, *Rubcn* Mut. TBI D3=6. Statistical analyses for all data: Two-way ANOVA with post hoc Tukey's test. Significance is represented by * P-value<0.05, ** P-value<0.005, *** P-value<0.0005, **** P-value<0.0001.

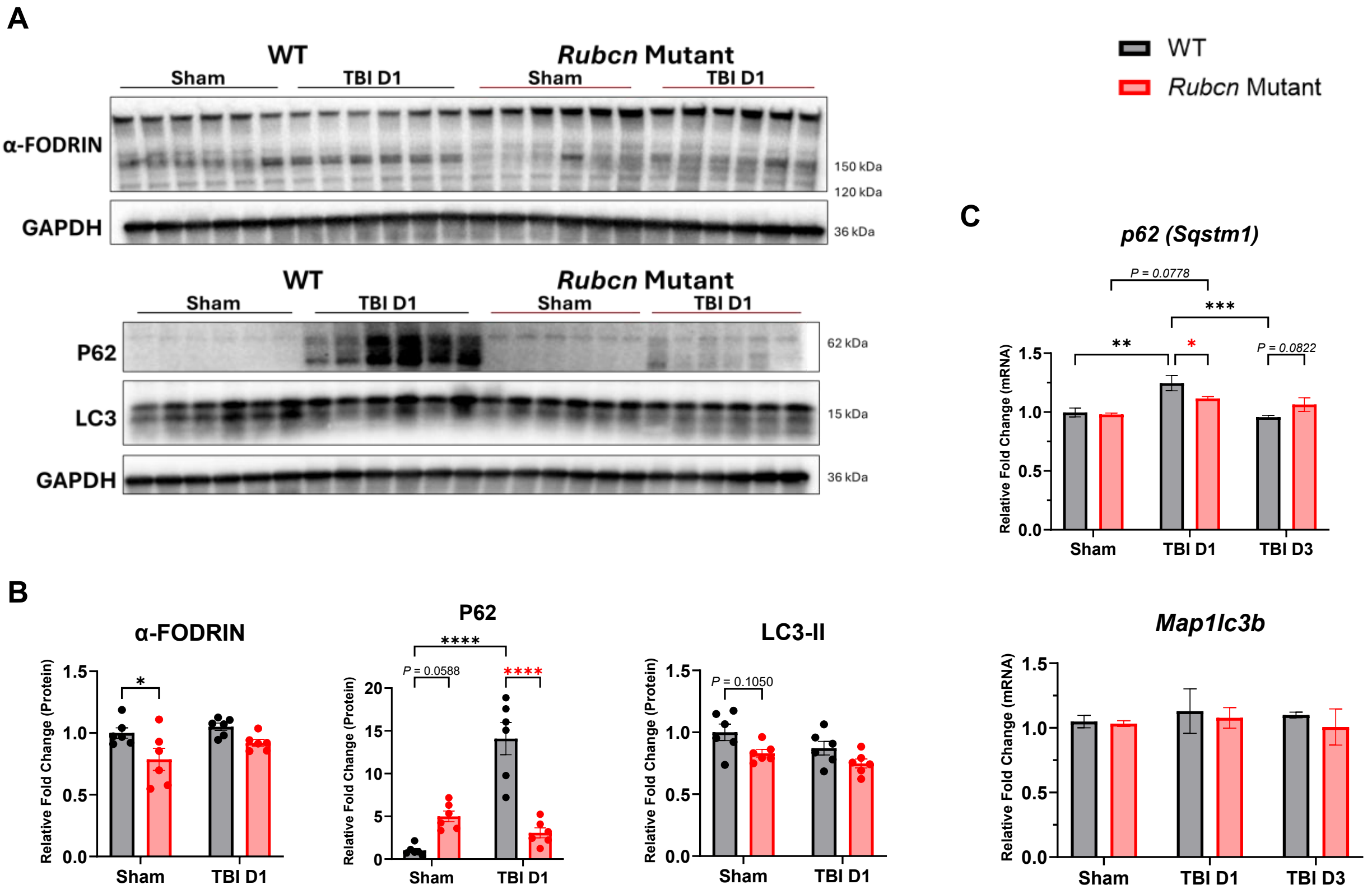

Supplemental Figure 2: Rubicon mutation decreases injury-induced autophagy inhibition. **A** Immunoblot comparing damage-associated marker (top) and autophagy markers (bottom) in the ipsilateral cortices from wild-type and *Rubcn*-mutant mice at 1 dpi to sham. **B** Densitometric quantification of immunoblot in **A**. **C** Fold change in gene expression levels of *Sqstm1* and *Map1lc3b* in wild-type and *Rubcn*-mutant mice at 1 and 3 dpi compared to sham. All bars represent mean ± s.e.m. Sample size (*n*) for **A-B** were as follows: WT Sham=6, WT TBI D1=6, *Rubcn* Mut. Sham=6, *Rubcn* Mut. D1=6. Sample size (*n*) for **C** were as follows: WT Sham=3, WT TBI D1=3, WT TBI D3=3, *Rubcn* Mut. Sham=3, *Rubcn* Mut. D1=3, *Rubcn* Mut. D3=3. Statistical analyses for all data: Two-way ANOVA with post hoc Tukey's test and significance is represented by * *P*-value<0.05, ** *P*-value<0.005, *** *P*-value<0.0005, **** *P*-value<0.0001.

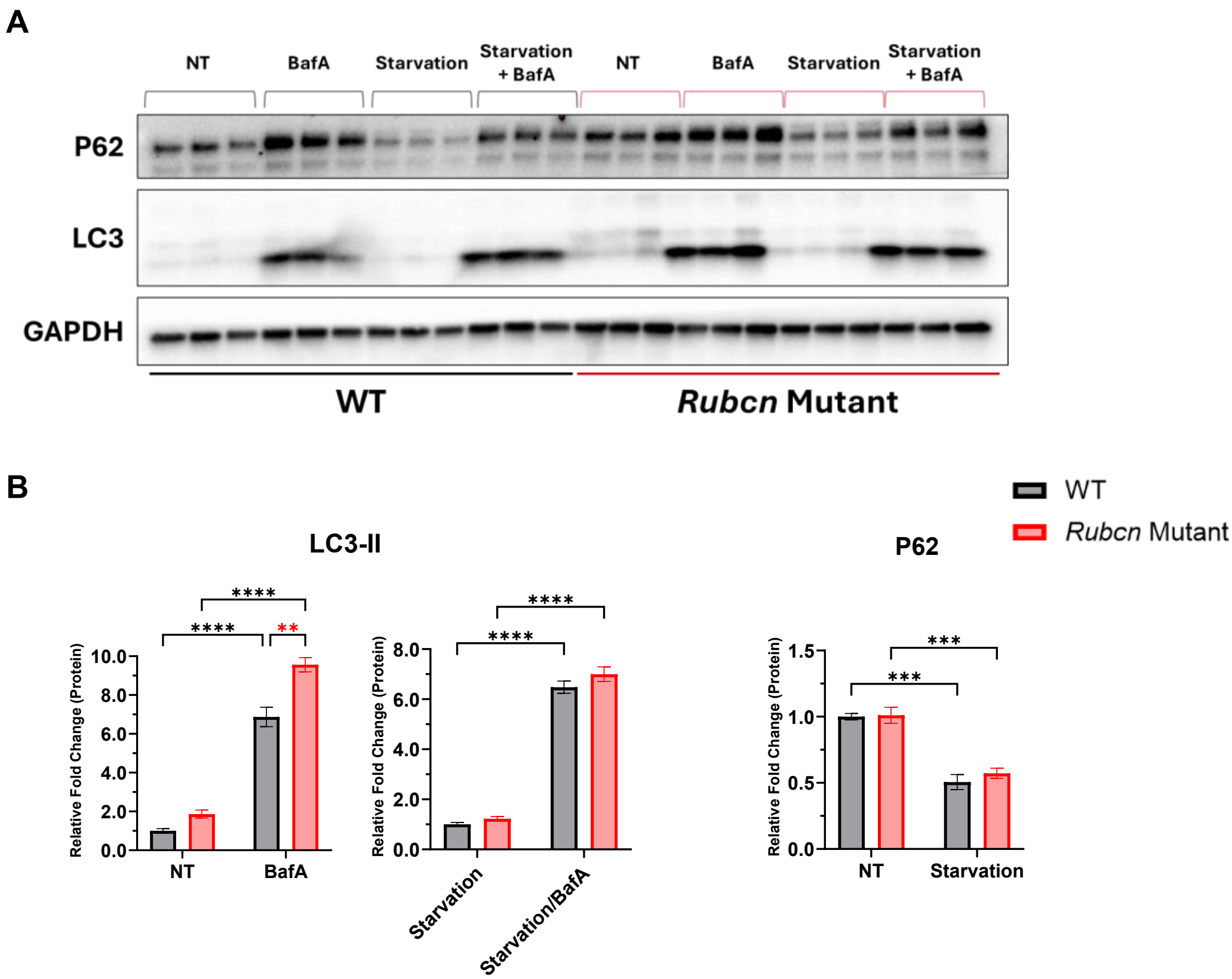

Supplemental Figure 3: Rubicon mutation increases baseline autophagy flux *in vitro.*
A Immunoblot of wild-type and *Rubcn*-mutant bone marrow derived macrophages (BMDMs) pre-treated with 50 nM bafilomycin A1 (BafA) for an hour (h) and/or serum-starved (Starvation) for 4 h. NT represents no treatment group. **B** Densitometric analysis of immunoblot in **A**. Data represent mean ± s.e.m. Sample size (*n*) were as follows: *n*=3 mice per group. Statistical analyses: Two-way ANOVA with post hoc Tukey's test and significance represented by * *P*-value<0.05, ** *P*-value<0.005, *** *P*-value<0.0005, *** *P*-value<0.0001.

Supplemental Figure 4

A

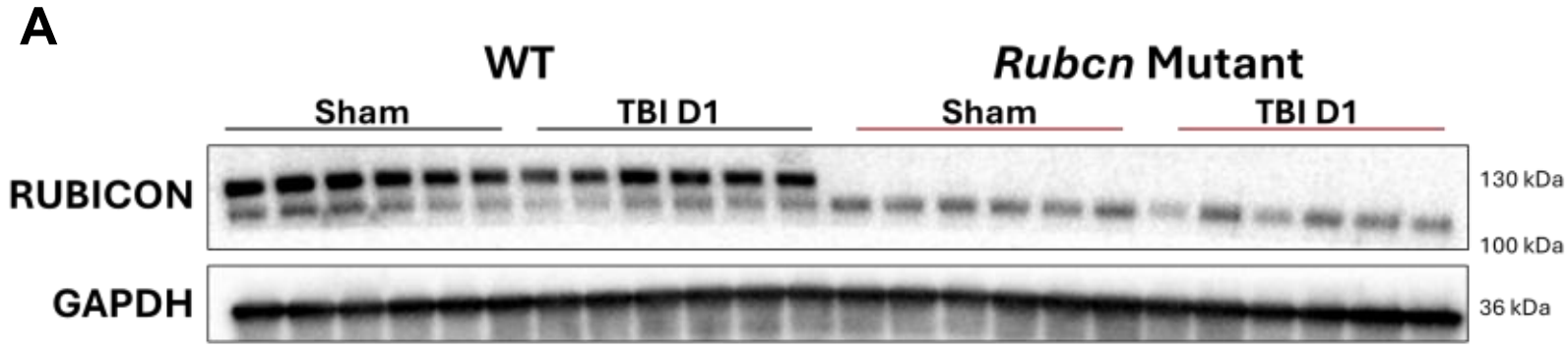

B

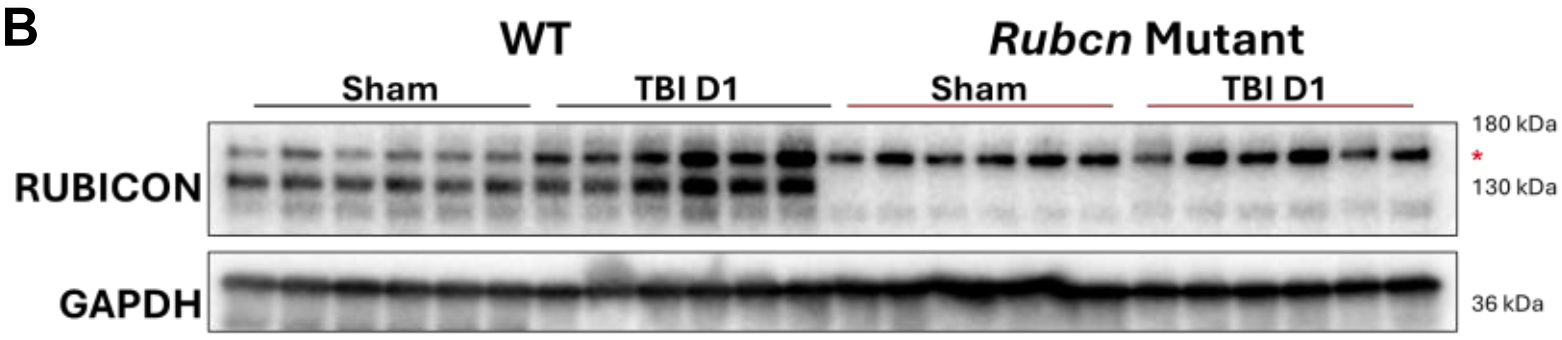

C

Peptide	Positions in Master Proteins	WT1RbA	WT2RbA	WT1RbB	WT2RbB	MUT1RbA	MUT2RbA	MUT1RbB	MUT2RbB
**p1**	Q80U62 [35-51]	757897.12							
**p2**	Q80U62 [79-87]	901011.991	478468.75	297272.9	647483.29				
**p3**	Q80U62 [103-120]	1533532.72	649298.06	552695.3	503595.54				
**p4**	Q80U62 [195-204]	589246.797							
**p5**	Q80U62 [248-264]	515630.44	189056.61		134008.99				
**p6**	Q80U62 [364-378]	1367169.48	1044234.4	907214	772475.59	350636.6	804352.7	1182071	908300
**p7**	Q80U62 [389-404]	740187.94		408680.6	429816.09			2138834	548258.7
**p8**	Q80U62 [550-560]	843720.199	275065.28	353562	131211.96	71575.27		1193284	167100.9
**p9**	Q80U62 [620-644]		234036.55		183236.68			782318.1	178607.3
**p10**	Q80U62 [645-666]	2176758	813405.06	1241401	822679.16			1955368	1109239
**p11**	Q80U62 [768-785]		54991.824	125890.1	62090.446		31876.57	162106.6	70755.47
**p12**	Q80U62 [872-892]								

D

Accession	Description	Gene Symbol	WT RbA /IgG	WT RbB /IgG	MUT RbA /IgG	MUT RbB /IgG	WT/MUT RbA	WT/MUT RbB	Average WT/MUT
Q8BMT4	Transforming growth factor beta activator LRRC33 [OS=Mus musculus]	Nrros	5.40	3.07	1.23	0.16	4.37	18.72	11.54
Q8K245	UV radiation resistance-associated protein [OS=Mus musculus]	Uvrag	64.64	51.67	9.47	79.07	6.83	0.65	3.74
Q8VD65	Phosphoinositide 3-kinase regulatory subunit 4 [OS=Mus musculus]	Pik3r4	9.08	8.47	1.89	9.10	4.81	0.93	2.87
O88597	Beclin-1 [OS=Mus musculus]	Becn1	5.49	5.34	1.67	6.68	3.30	0.80	2.05
Q6PF93	Phosphatidylinositol 3-kinase catalytic subunit type 3 [OS=Mus musculus]	Pik3c3	2.17	1.78	0.73	1.93	2.99	0.92	1.96
Q6VNB8	WD repeat and FYVE domain-containing protein 3 [OS=Mus musculus]	Wdfy3	10.89	7.89	21.11	8.44	0.52	0.94	0.73

E

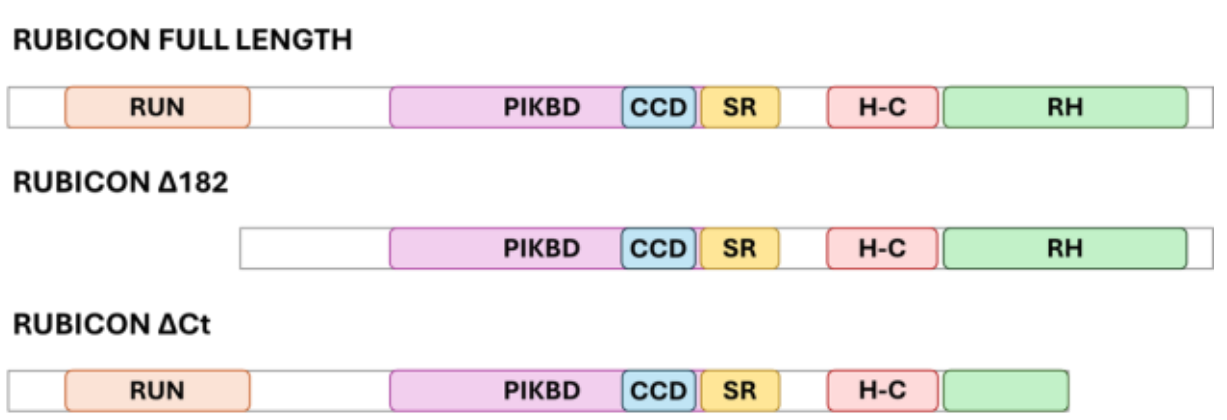

G

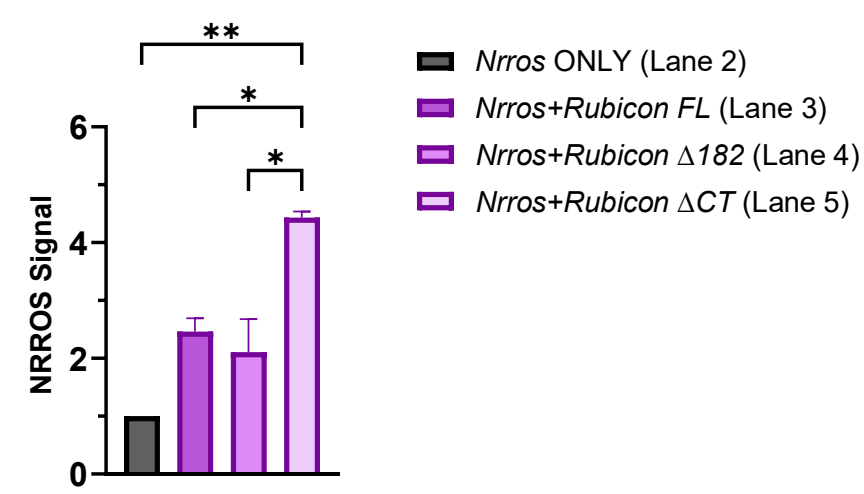

F

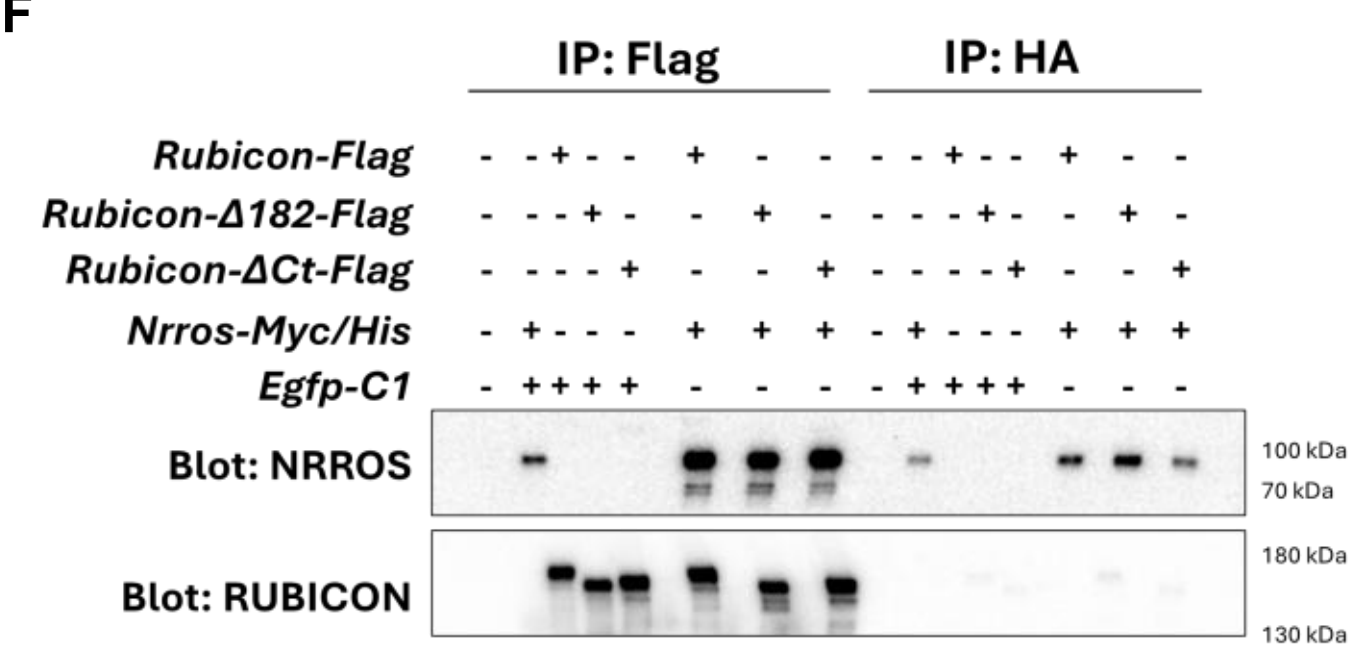

H

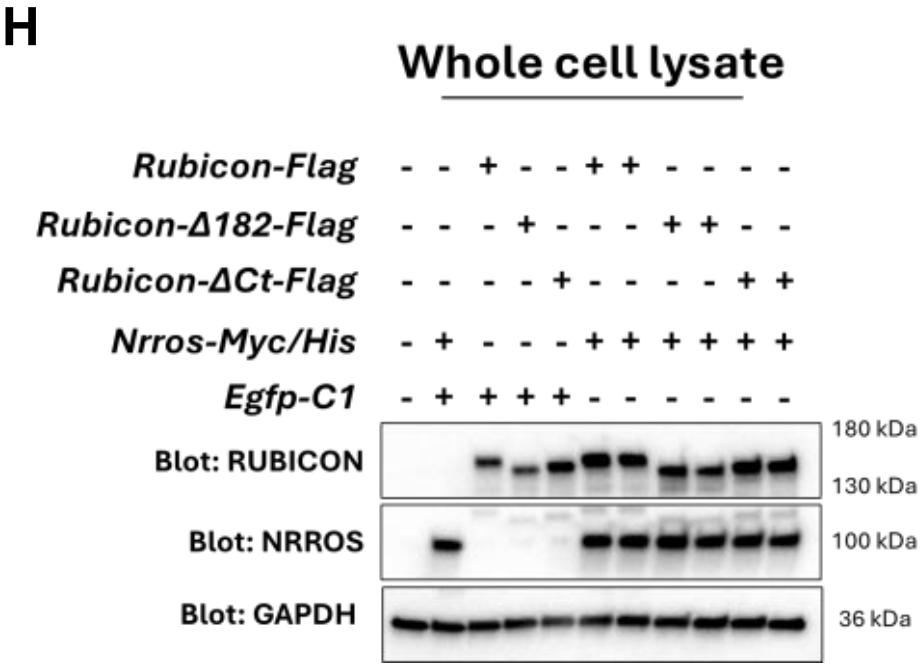

Supplemental Figure 4: Truncated RUBCN protein is present in *Rubcn*-mutant mice.
A Immunoblot comparing lysates of ipsilateral cortices from wild-type and *Rubcn*-mutant mice at 1 dpi to sham probed with Rubicon A (RbA) antibody. **B** Immunoblot comparing lysates of ipsilateral cortices from wild-type and *Rubcn*-mutant mice at 3 dpi to sham probed with Rubicon B (RbB) antibody. * represents background signal. **C** Table showing normalized peptide abundance queried for peptides p1 to p12 across the length of RUBCN protein in wild-type and *Rubcn*-mutant immunoprecipitates using RbA and RbB antibodies. Sample size (*n*) = 2 biological replicates for each genotype. **D** Fold change of comparatively enriched proteins in wild-type over *Rubcn*-mutant immunoprecipitates using RbA and RbB antibodies. **E** Schematic diagram showing full-length and truncated mutants of RUBCN proteins and their protein domains. PIKBD: PI3K binding domain, CCD: coiled-coil domain, SR: serine-rich domain, H-C: helix-coil-rich domain, RH: Rubicon homology domain. **F** Immunoprecipitation assay showing lysates of HEK293T cells transfected with indicated plasmids for 24 h and immunoprecipitated with anti-FLAG or anti-HA agarose beads. Immunoprecipitates were subjected to immunoblot with the indicated antibodies. **G** Fold change of NRROS signal in *Nrros*-Myc/His and EGFP-*Rubicon (full-length (FL), ∆182, or ∆CT*)-Flag co-transfected cells to EGFP-C1 and *Nrros*-Myc/His (*Nrros* ONLY) co-transfected cells. HA IP is used as negative control. Each FLAG IP sample was normalized to corresponding to HA IP sample. Data represent mean ± s.e.m. Sample size (*n*) = 2 biological replicates for EGFP-*Rubicon(FL, ∆182, or ∆CT*)-Flag and *Nrros*-Myc/His co-transfected cells. Statistical analysis: One-way ANOVA with post hoc Tukey's test and significance represented by * *P*-value<0.05, ** *P*-value<0.005. **H** Immunoblot of whole cell lysates used for immunoprecipitation assay in **F**.
