# Supplementary material for: Rubicon modulates neuroimmune responses following traumatic brain injury
Source: Cell Death Dis. 2026 Jul 27;17(1):899. doi: 10.1038/s41419-026-09083-4 (PMC13687110; doi:10.1038/s41419-026-09083-4)

4A

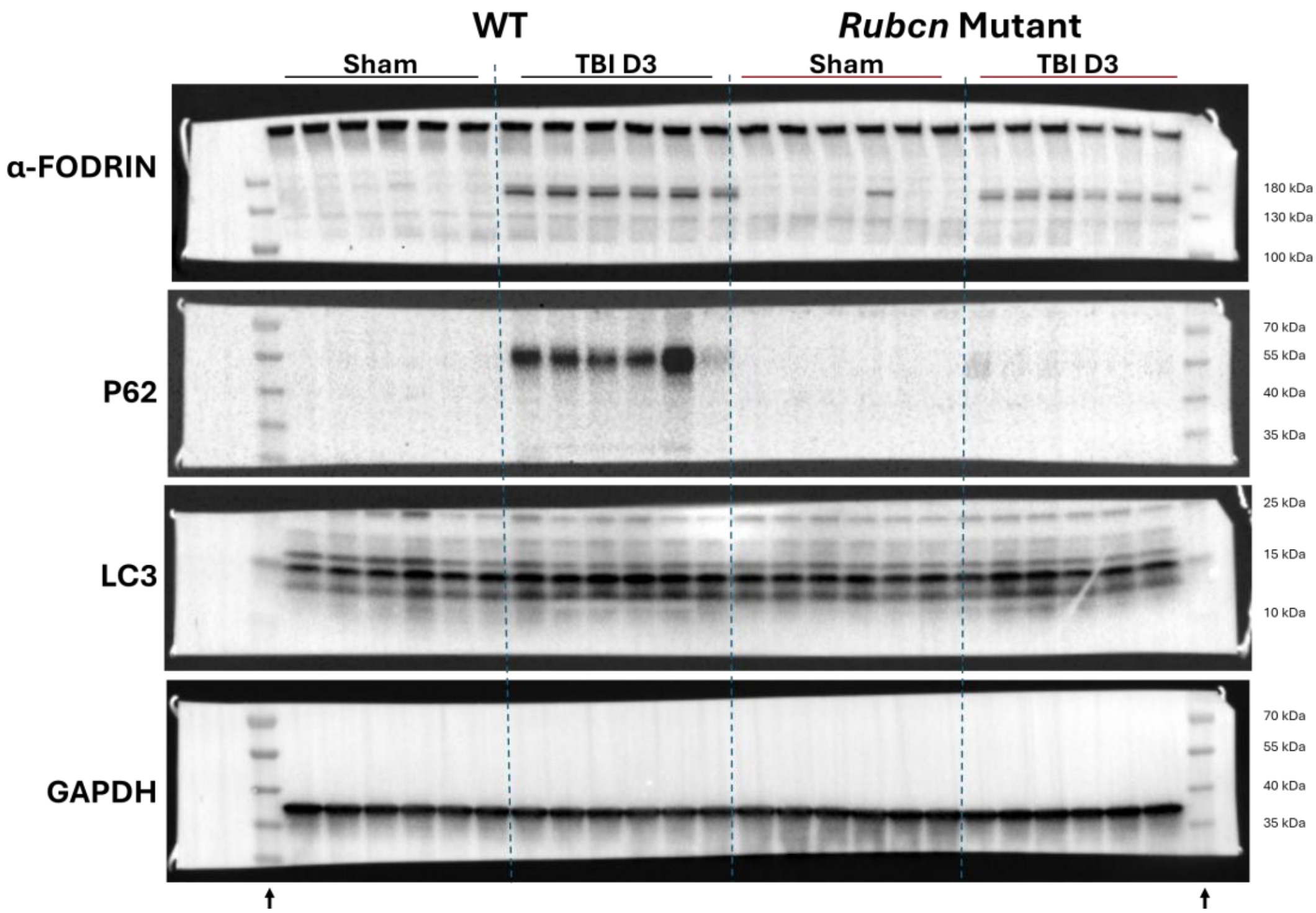
WT
Rubcn Mutant
Sham
TBI D3
Sham
TBI D3
α-FODRIN
P62
LC3
GAPDH
180 kDa
130 kDa
100 kDa
70 kDa
55 kDa
40 kDa
35 kDa
25 kDa
15 kDa
10 kDa
70 kDa
55 kDa
40 kDa
35 kDa

4C

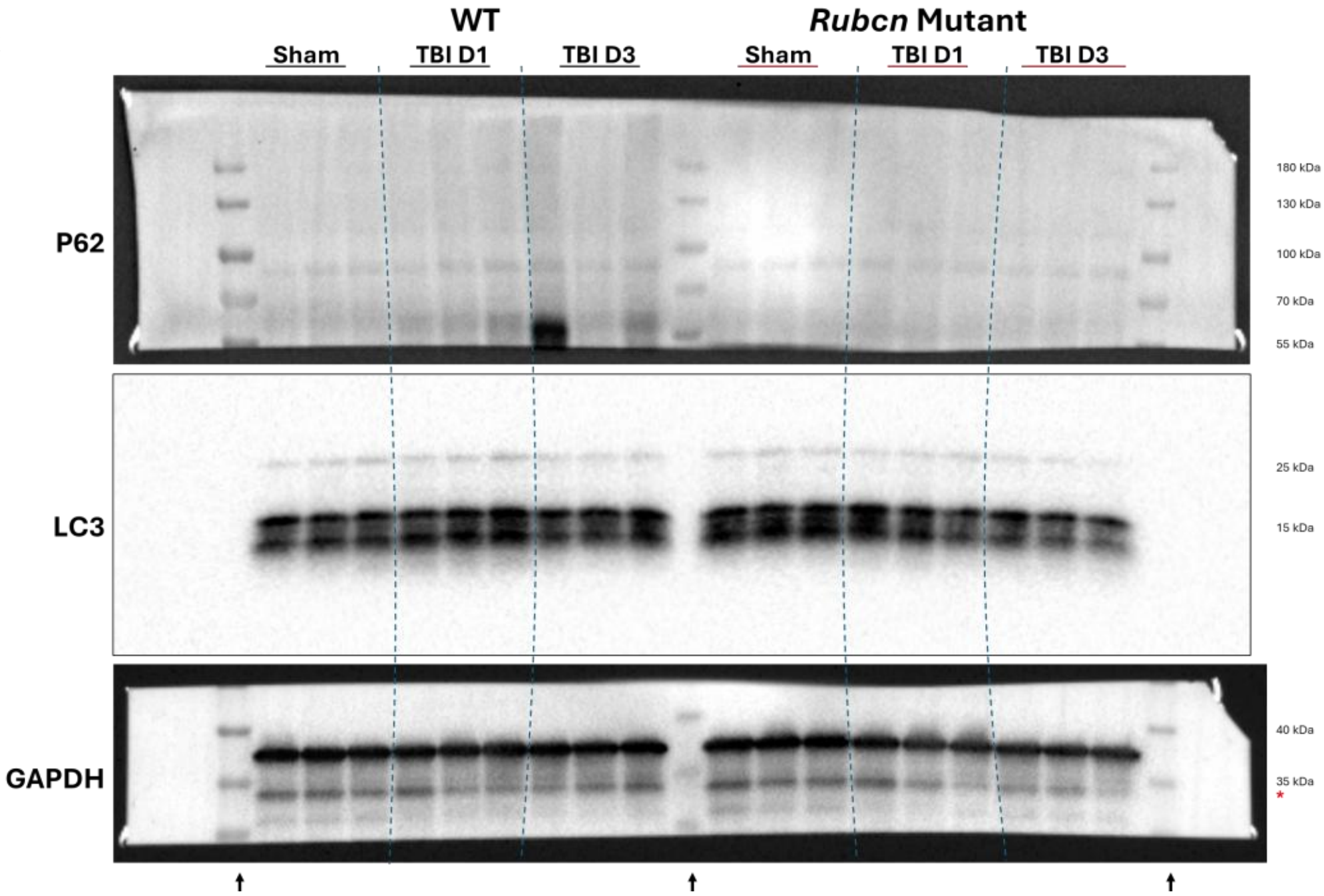
WT
Rubcn Mutant
Sham
TBI D1
TBI D3
Sham
TBI D1
TBI D3
P62
LC3
GAPDH
180 kDa
130 kDa
100 kDa
70 kDa
55 kDa
25 kDa
15 kDa
40 kDa
35 kDa
*

4E

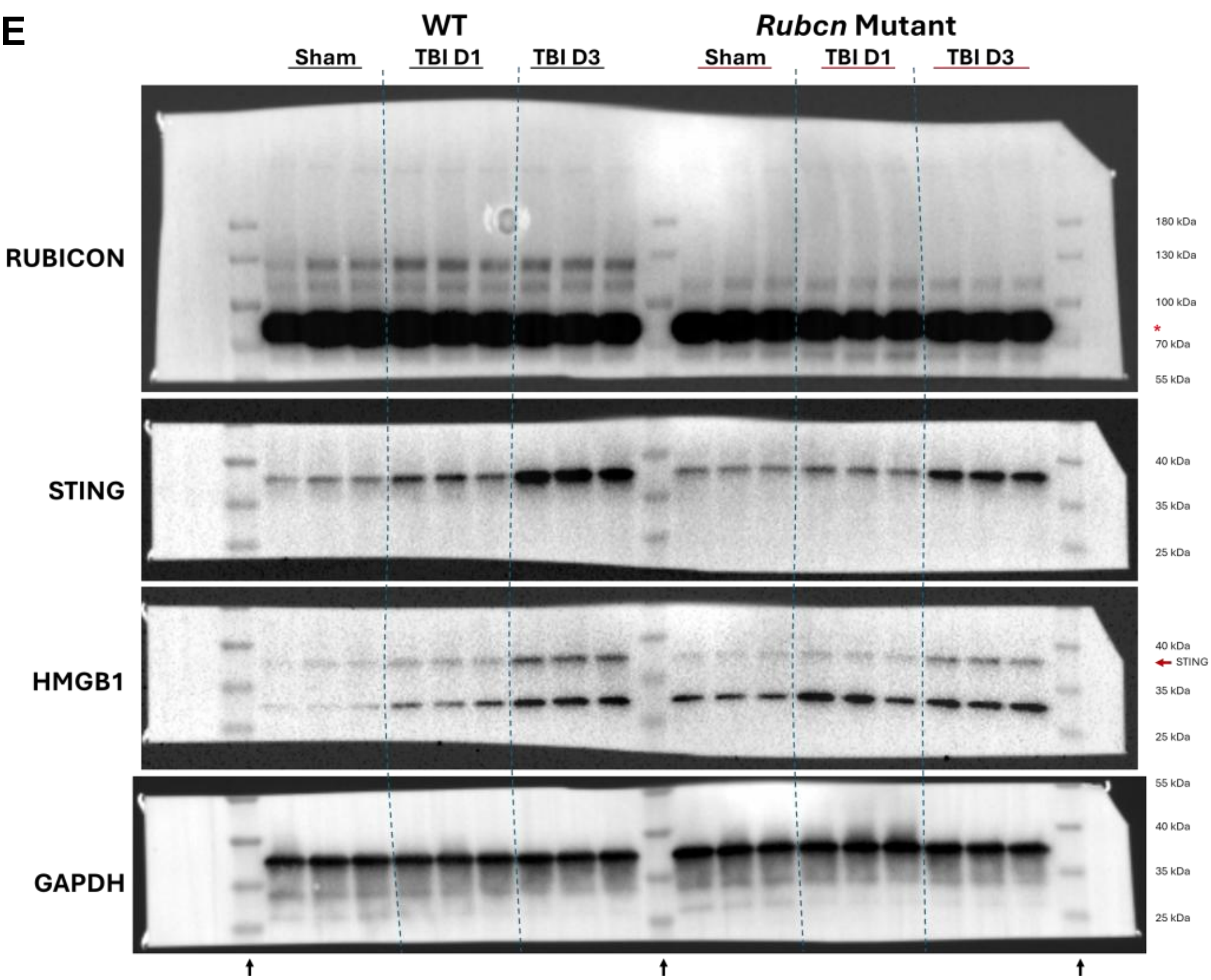
WT
Rubcn Mutant
Sham
TBI D1
TBI D3
Sham
TBI D1
TBI D3
RUBICON
STING
HMGB1
GAPDH
180 kDa
130 kDa
100 kDa
*
70 kDa
55 kDa
40 kDa
35 kDa
25 kDa
40 kDa
STING
35 kDa
25 kDa
55 kDa
40 kDa
35 kDa
25 kDa

4C/E: TBI D1 All Samples

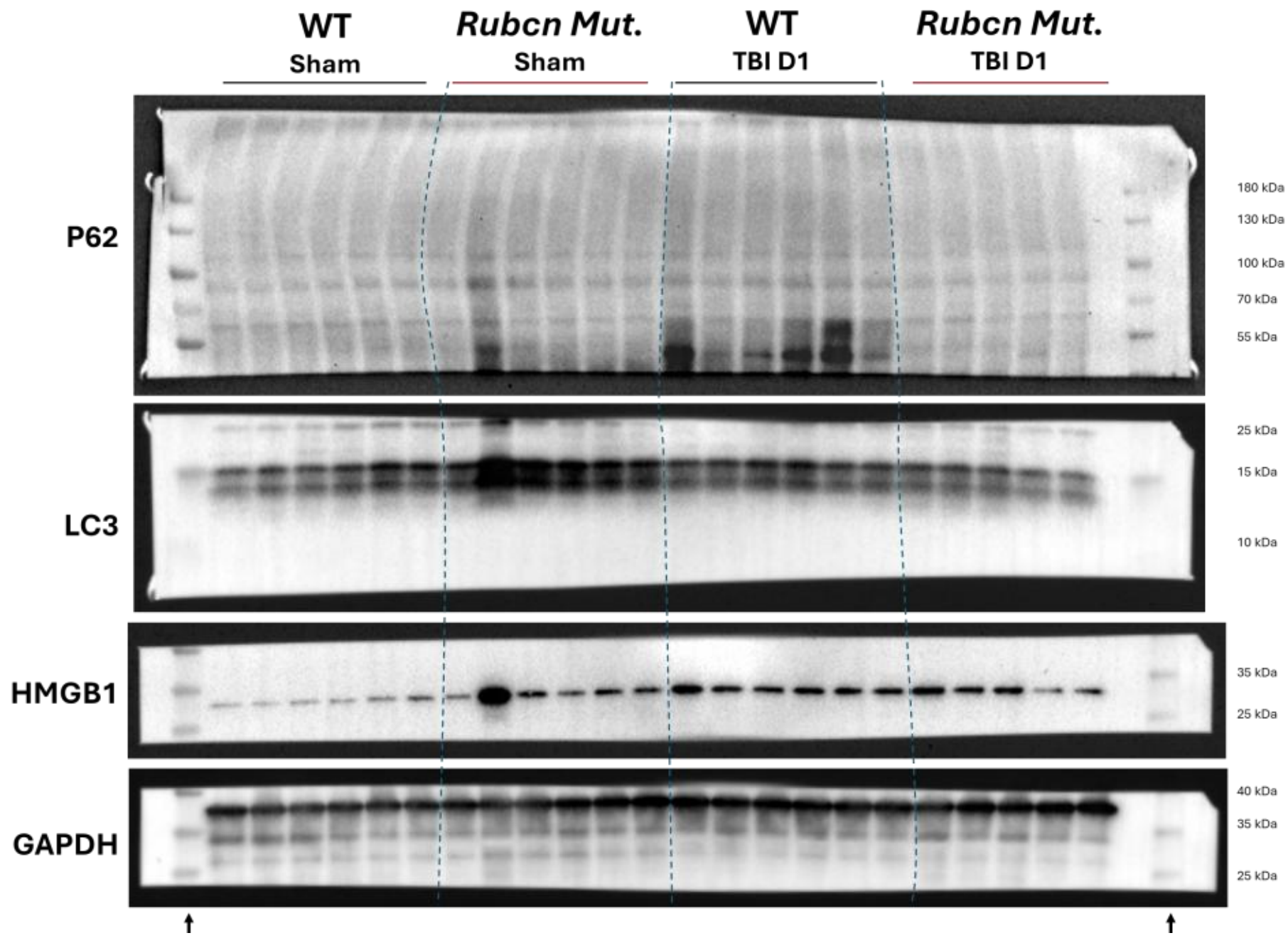

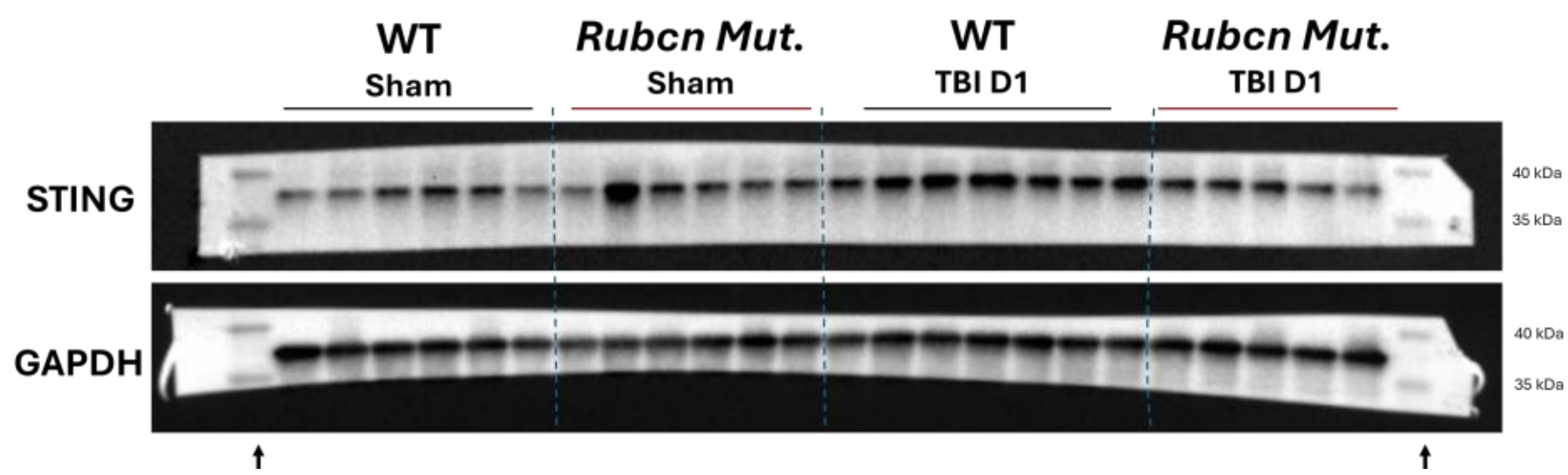

4C/E: TBI D3 All Samples

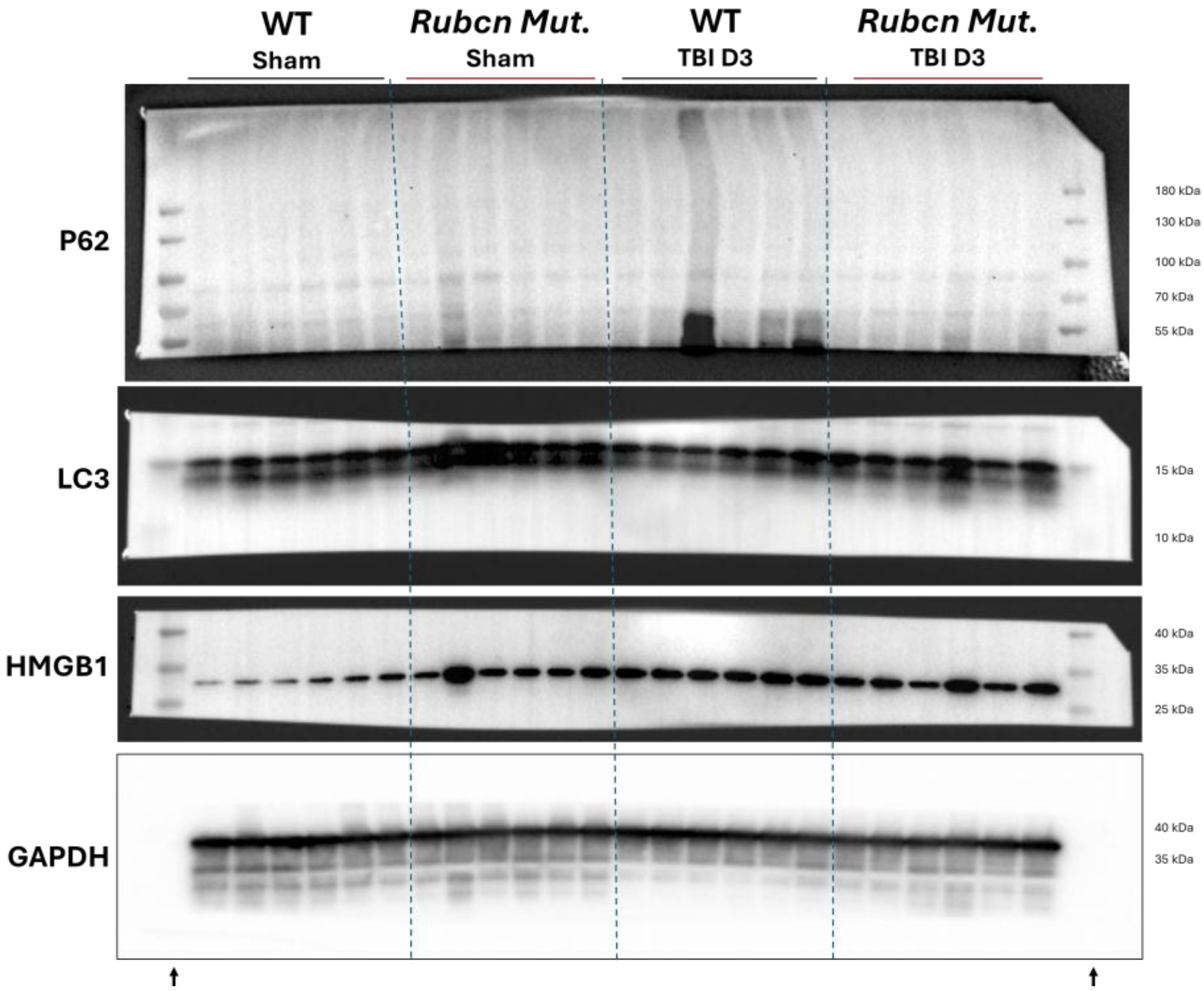

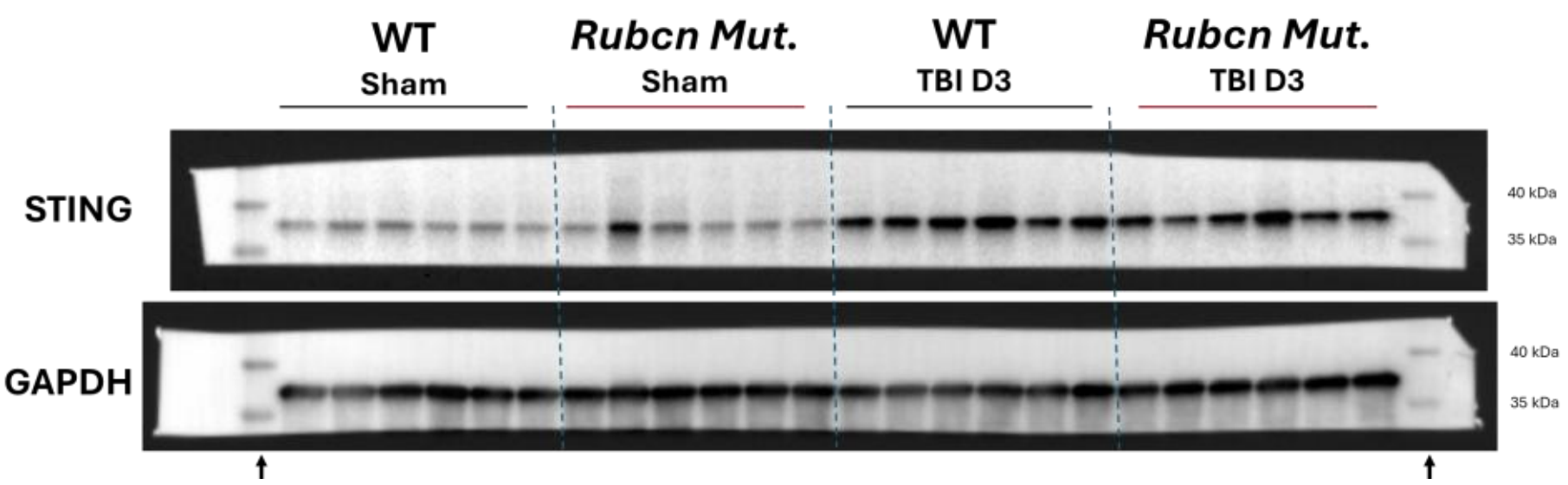

5A

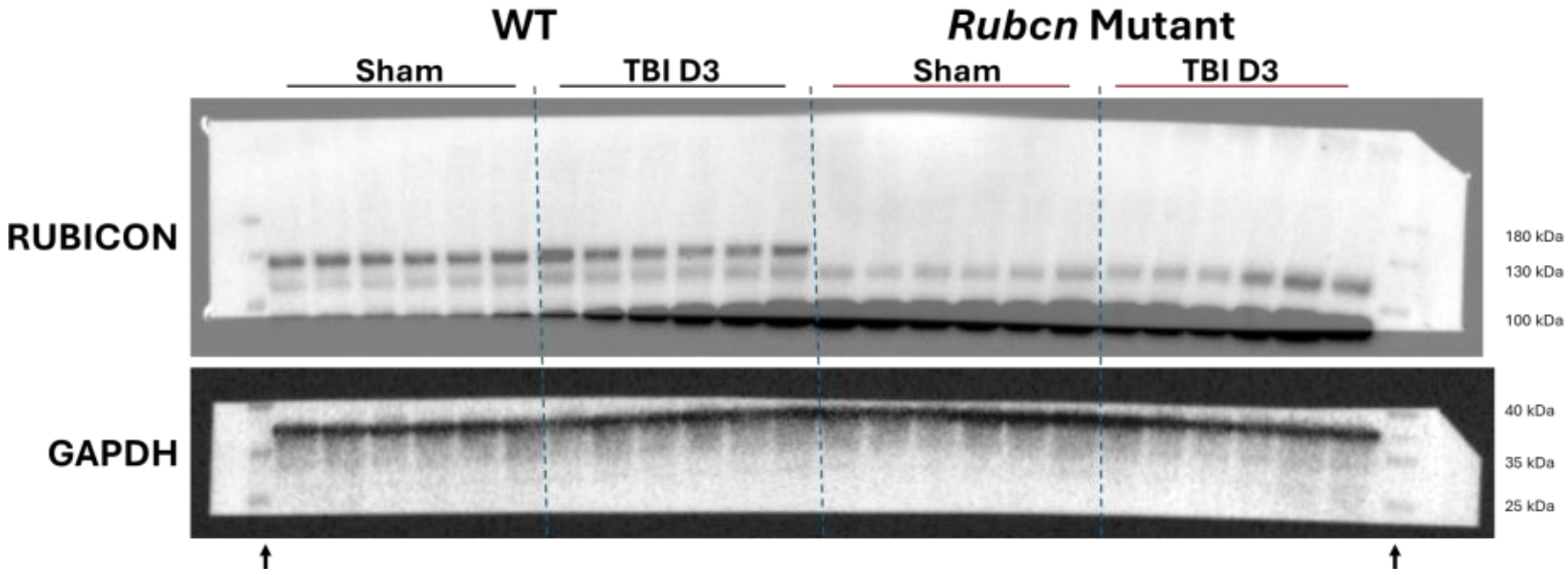

WT
Rubcn Mutant
Sham
TBI D3
Sham
TBI D3
RUBICON
GAPDH
180 kDa
130 kDa
100 kDa
40 kDa
35 kDa
25 kDa

5B

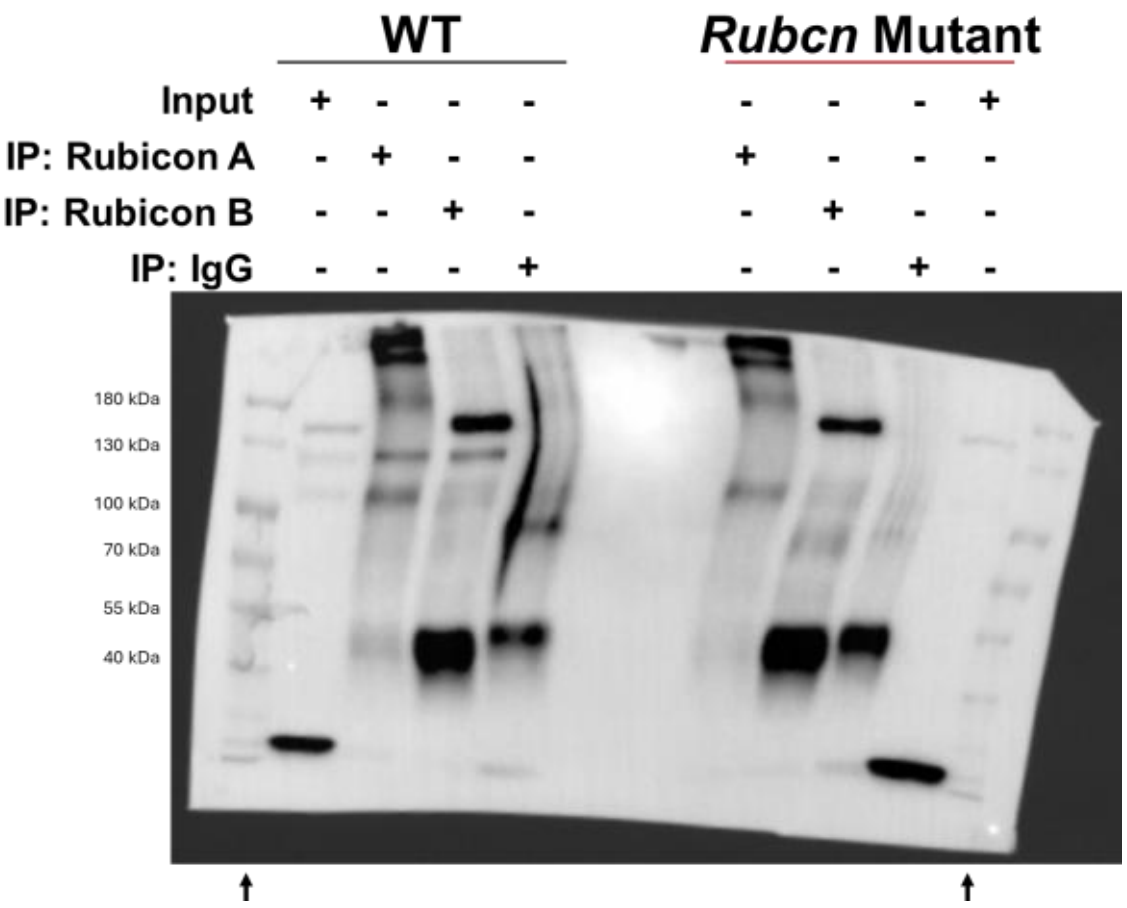

WT
Rubcn Mutant
Input
IP: Rubicon A
IP: Rubicon B
IP: IgG
180 kDa
130 kDa
100 kDa
70 kDa
55 kDa
40 kDa

5F

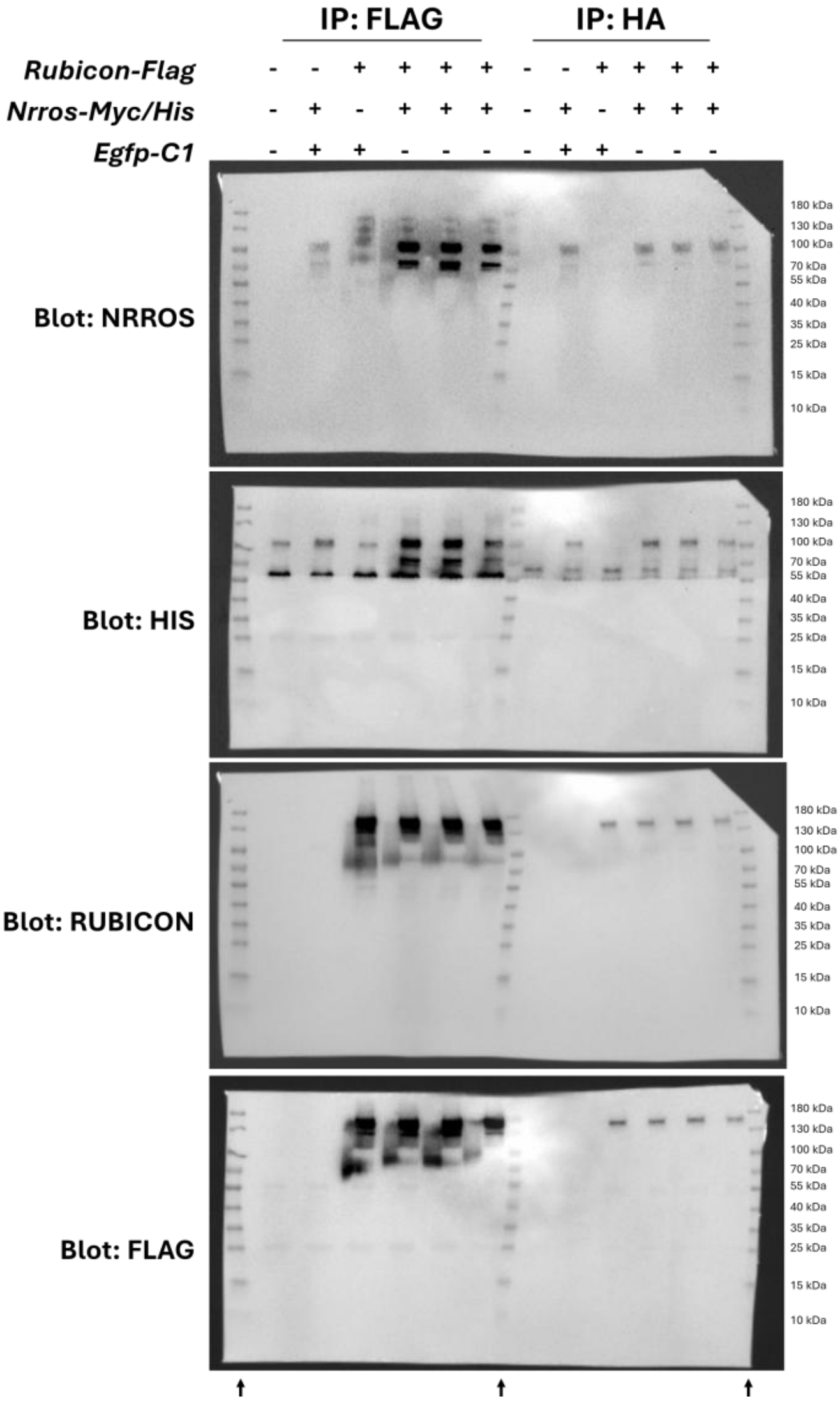

IP: FLAG
IP: HA
Rubicon-Flag
Nrros-Myc/His
Egfp-C1
Blot: NRROS
Blot: HIS
Blot: RUBICON
Blot: FLAG
180 kDa
130 kDa
100 kDa
70 kDa
55 kDa
40 kDa
35 kDa
25 kDa
15 kDa
10 kDa

5G

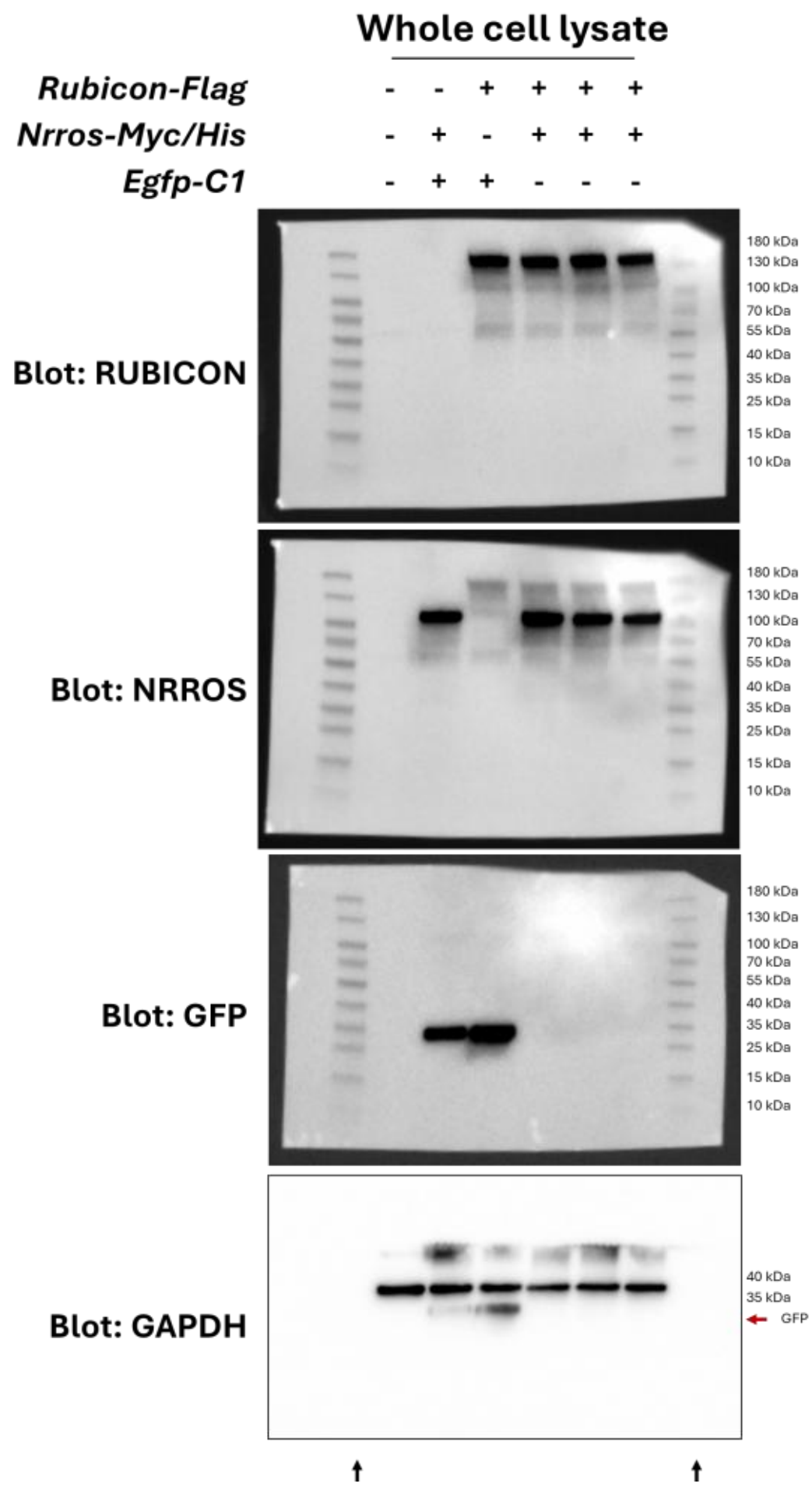

Whole cell lysate
Rubicon-Flag
Nrros-Myc/His
Egfp-C1
Blot: RUBICON
Blot: NRROS
Blot: GFP
Blot: GAPDH
180 kDa
130 kDa
100 kDa
70 kDa
55 kDa
40 kDa
35 kDa
25 kDa
15 kDa
10 kDa
GFP

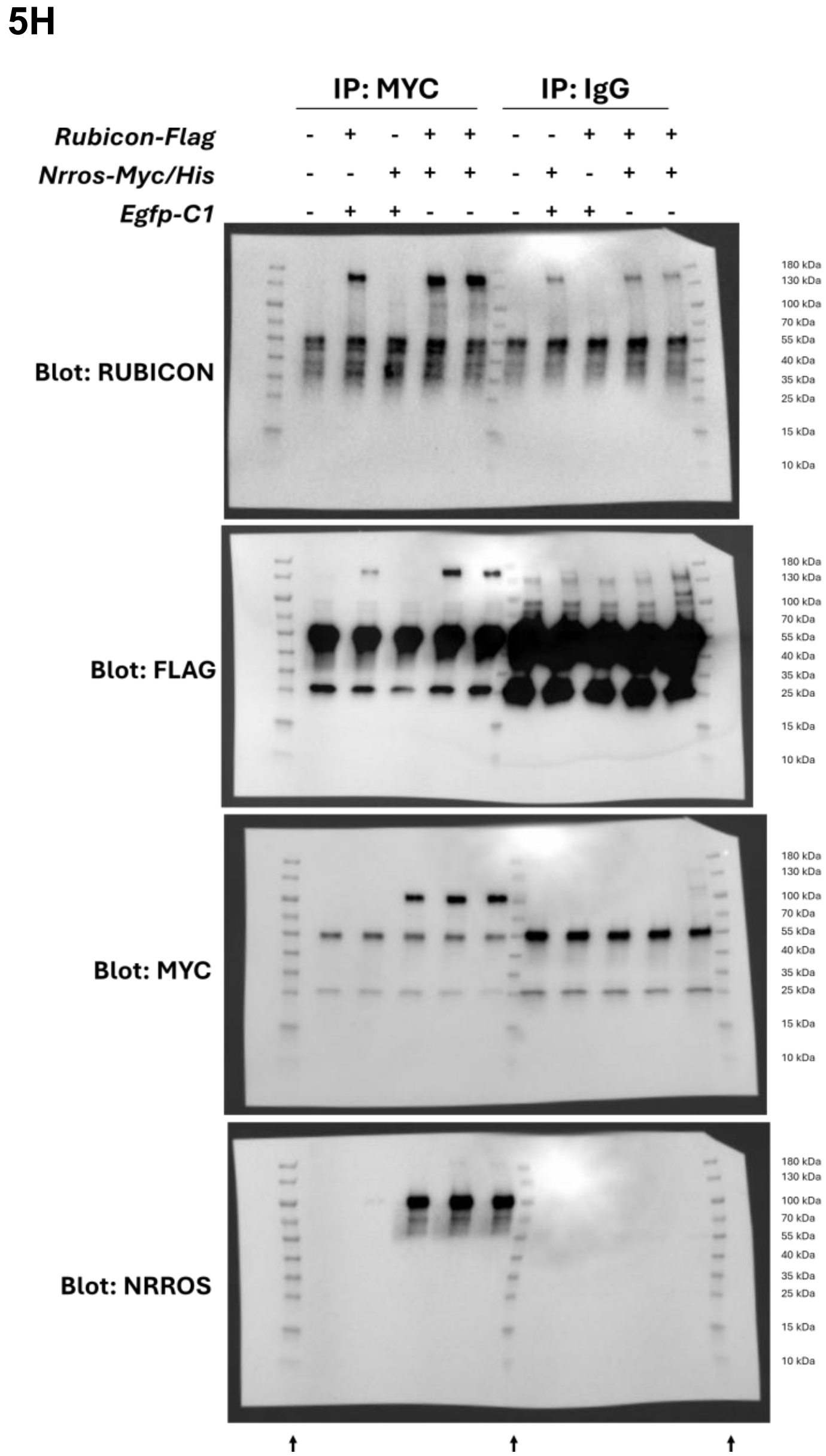
5H
IP: MYC
IP: IgG
Rubicon-Flag
Nrros-Myc/His
Egfp-C1
Blot: RUBICON
Blot: FLAG
Blot: MYC
Blot: NRROS
180 kDa
130 kDa
100 kDa
70 kDa
55 kDa
40 kDa
35 kDa
25 kDa
15 kDa
10 kDa

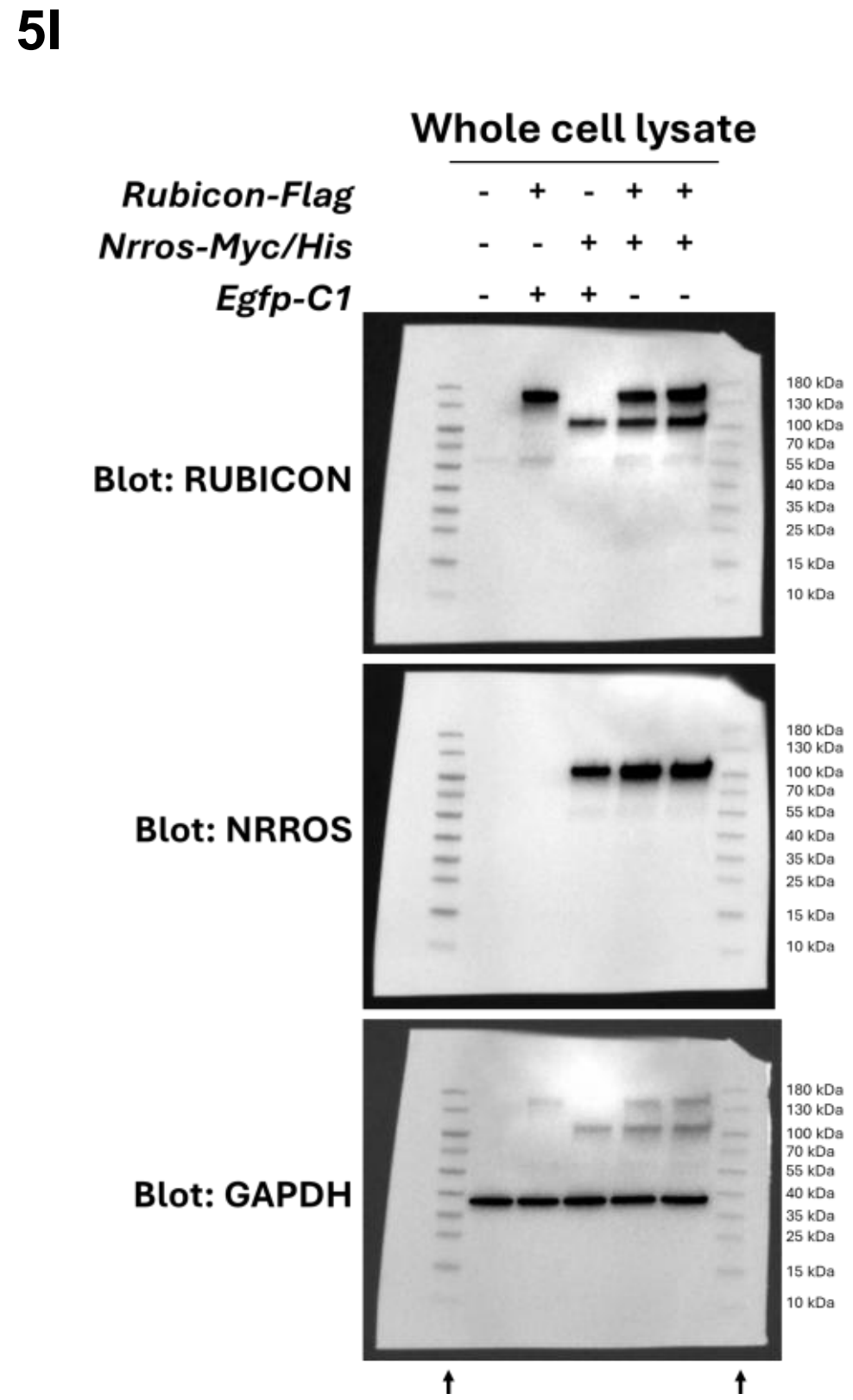
5I
Whole cell lysate
Rubicon-Flag
Nrros-Myc/His
Egfp-C1
Blot: RUBICON
Blot: NRROS
Blot: GAPDH
180 kDa
130 kDa
100 kDa
70 kDa
55 kDa
40 kDa
35 kDa
25 kDa
15 kDa
10 kDa

6A

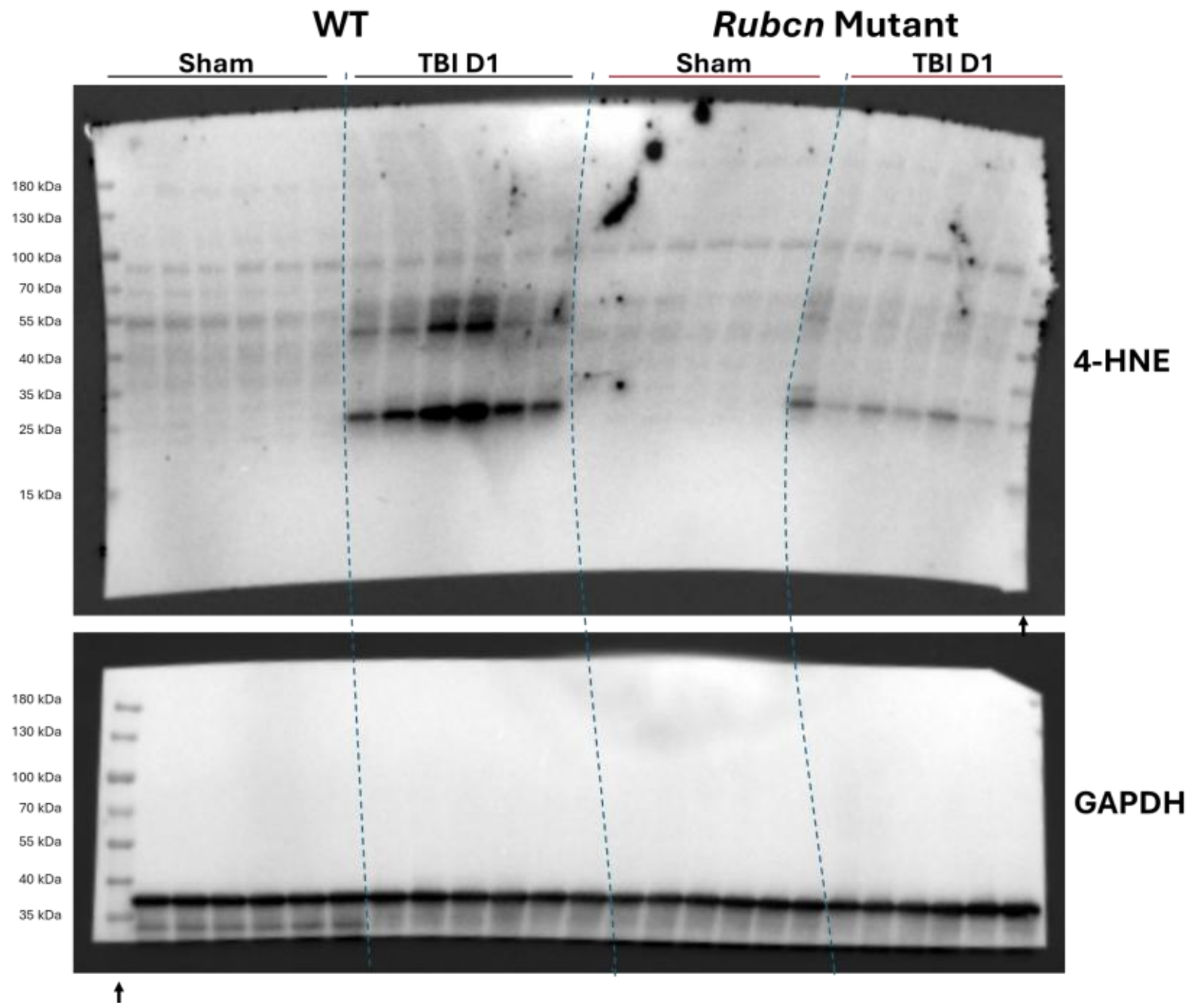
WT
Rubcn Mutant
Sham
TBI D1
Sham
TBI D1
180 kDa
130 kDa
100 kDa
70 kDa
55 kDa
40 kDa
35 kDa
25 kDa
15 kDa
4-HNE
180 kDa
130 kDa
100 kDa
70 kDa
55 kDa
40 kDa
35 kDa
GAPDH

6C

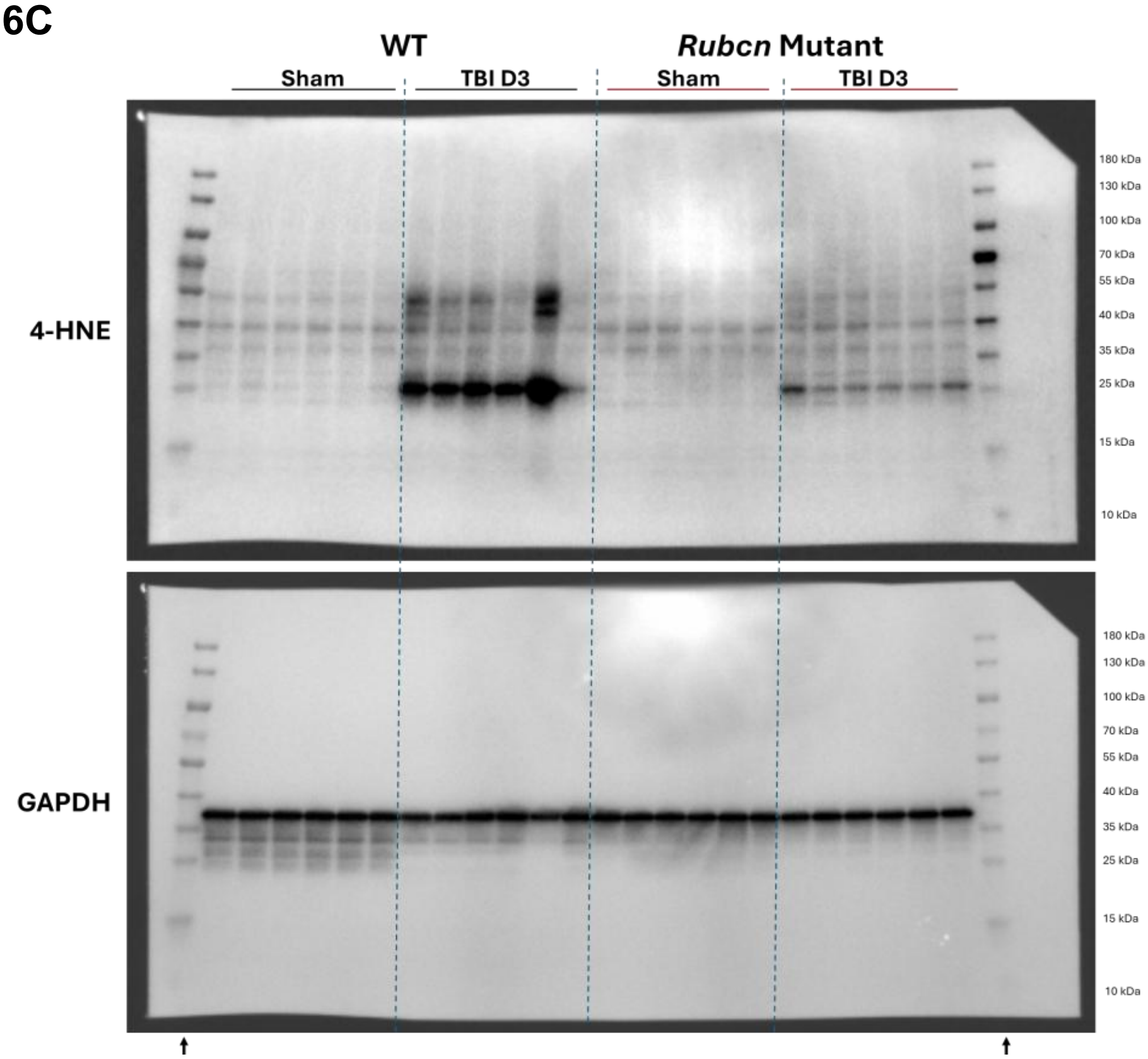
WT
Rubcn Mutant
Sham
TBI D3
Sham
TBI D3
4-HNE
180 kDa
130 kDa
100 kDa
70 kDa
55 kDa
40 kDa
35 kDa
25 kDa
15 kDa
10 kDa
GAPDH
180 kDa
130 kDa
100 kDa
70 kDa
55 kDa
40 kDa
35 kDa
25 kDa
15 kDa
10 kDa

S1C

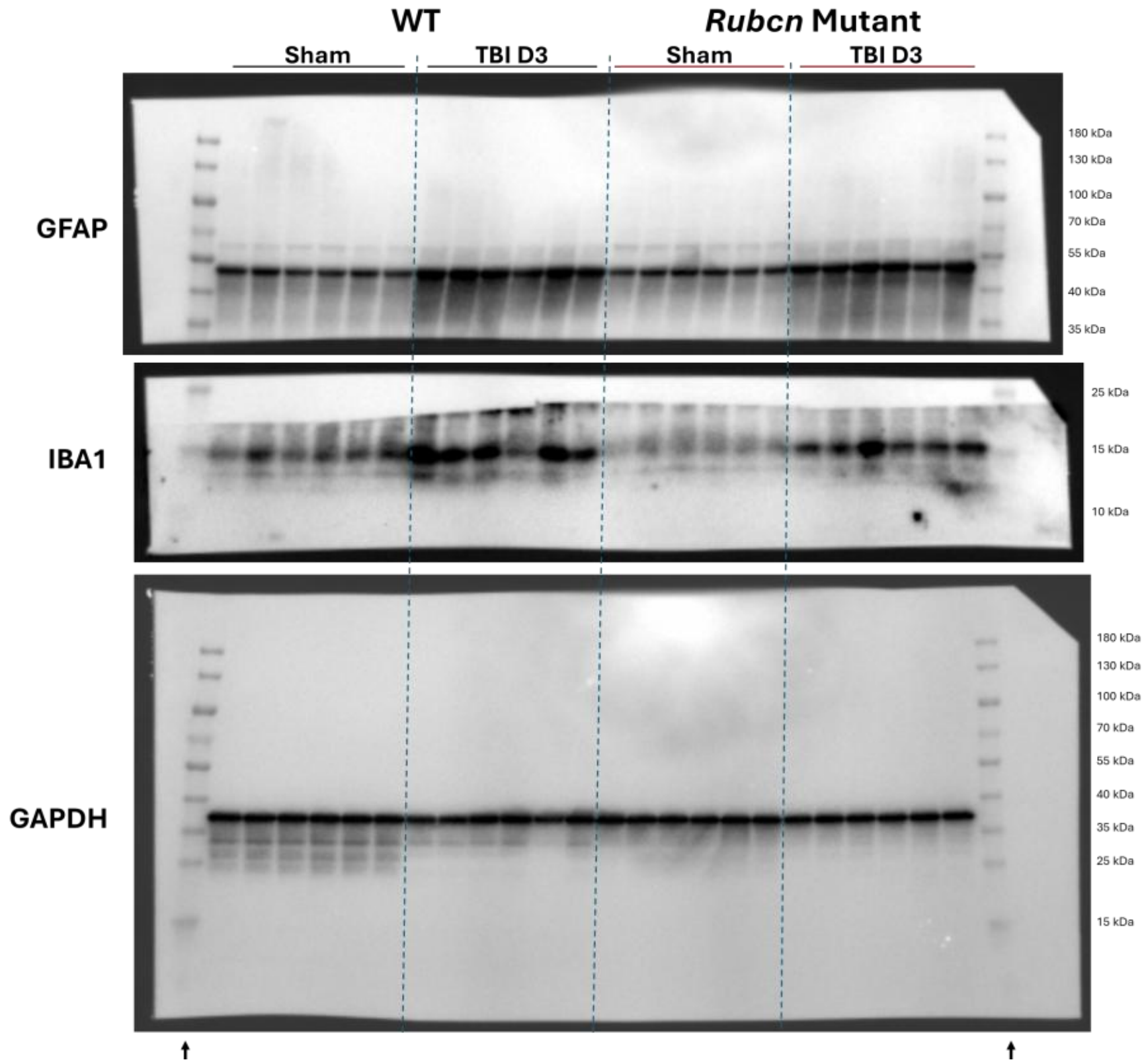
WT
Rubcn Mutant
Sham
TBI D3
Sham
TBI D3
GFAP
IBA1
GAPDH
180 kDa
130 kDa
100 kDa
70 kDa
55 kDa
40 kDa
35 kDa
25 kDa
15 kDa
10 kDa

S2A

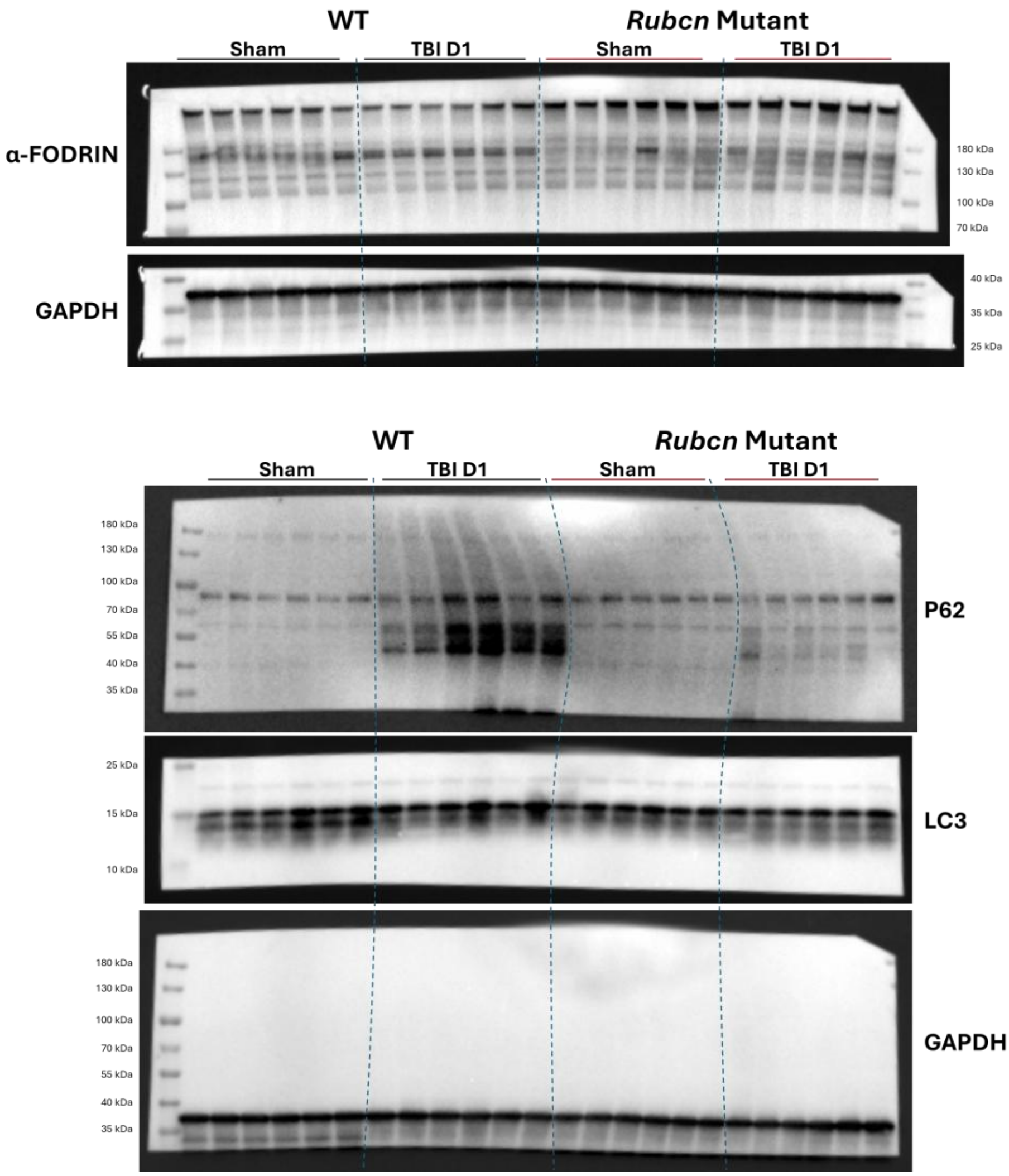
WT
Rubcn Mutant
Sham
TBI D1
Sham
TBI D1
α-FODRIN
180 kDa
130 kDa
100 kDa
70 kDa
GAPDH
40 kDa
35 kDa
25 kDa
WT
Rubcn Mutant
Sham
TBI D1
Sham
TBI D1
180 kDa
130 kDa
100 kDa
70 kDa
55 kDa
40 kDa
35 kDa
P62
25 kDa
15 kDa
10 kDa
LC3
180 kDa
130 kDa
100 kDa
70 kDa
55 kDa
40 kDa
35 kDa
GAPDH

S3A (1st replicate)

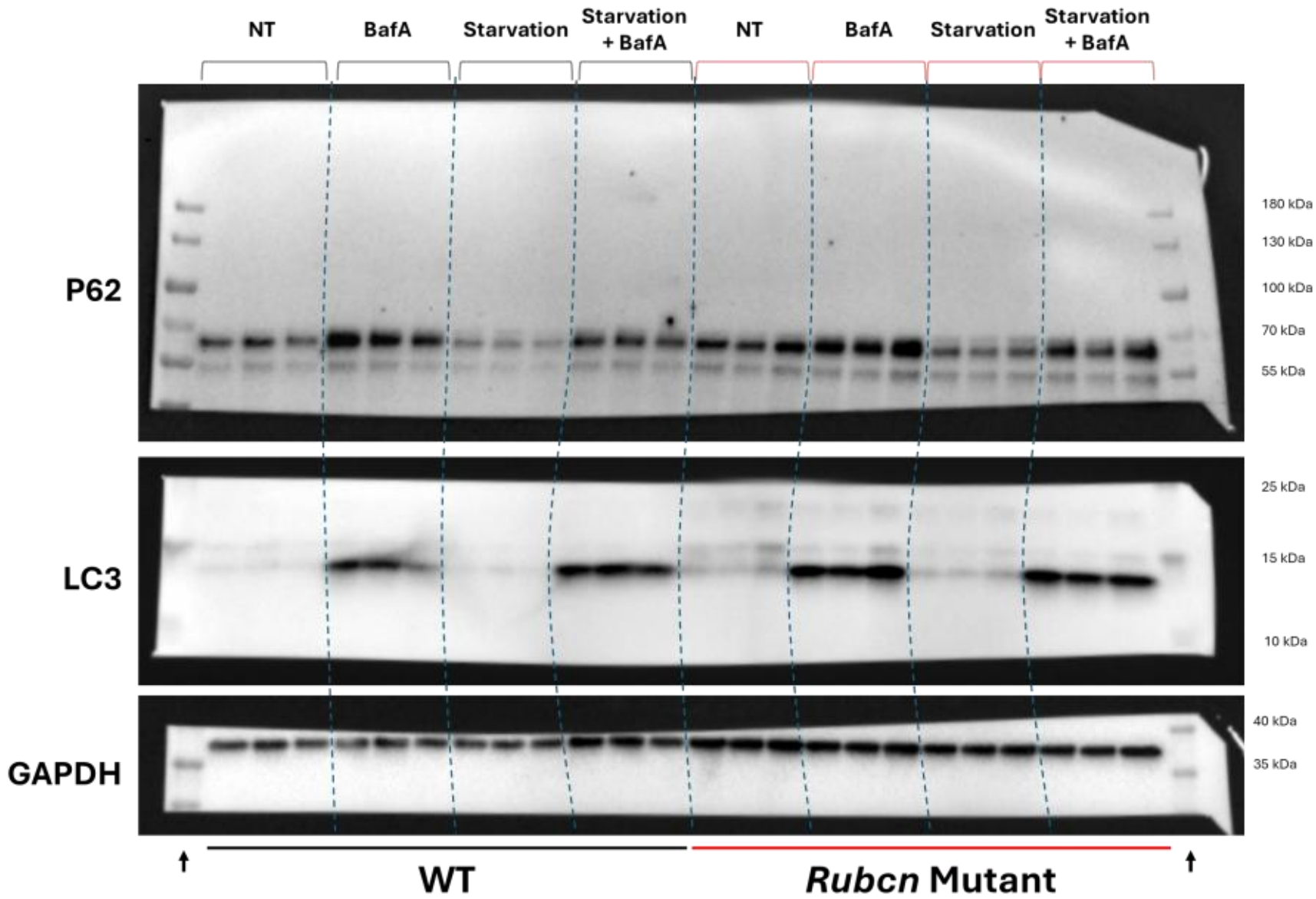

S3A (2nd replicate)

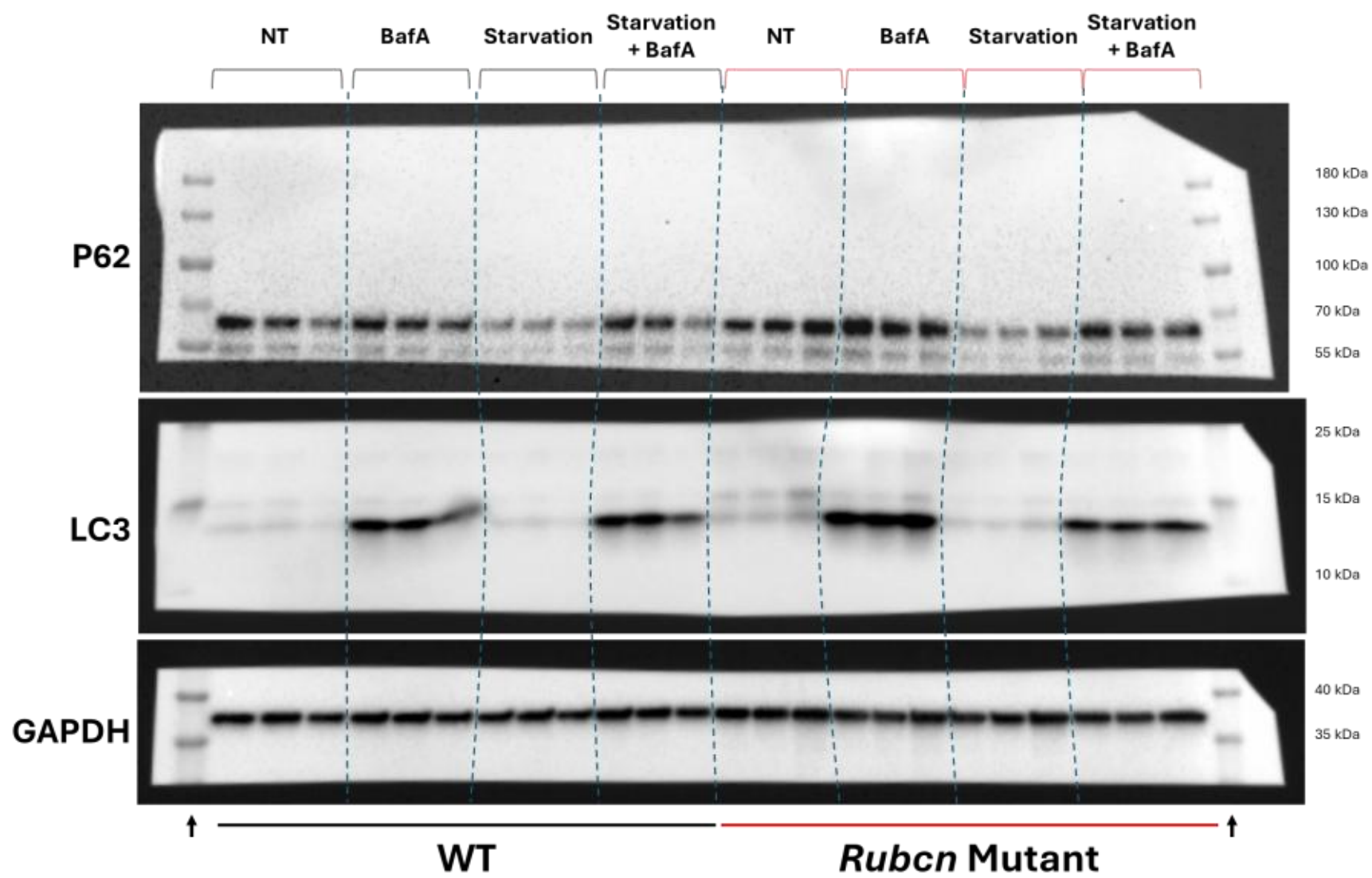

S4A

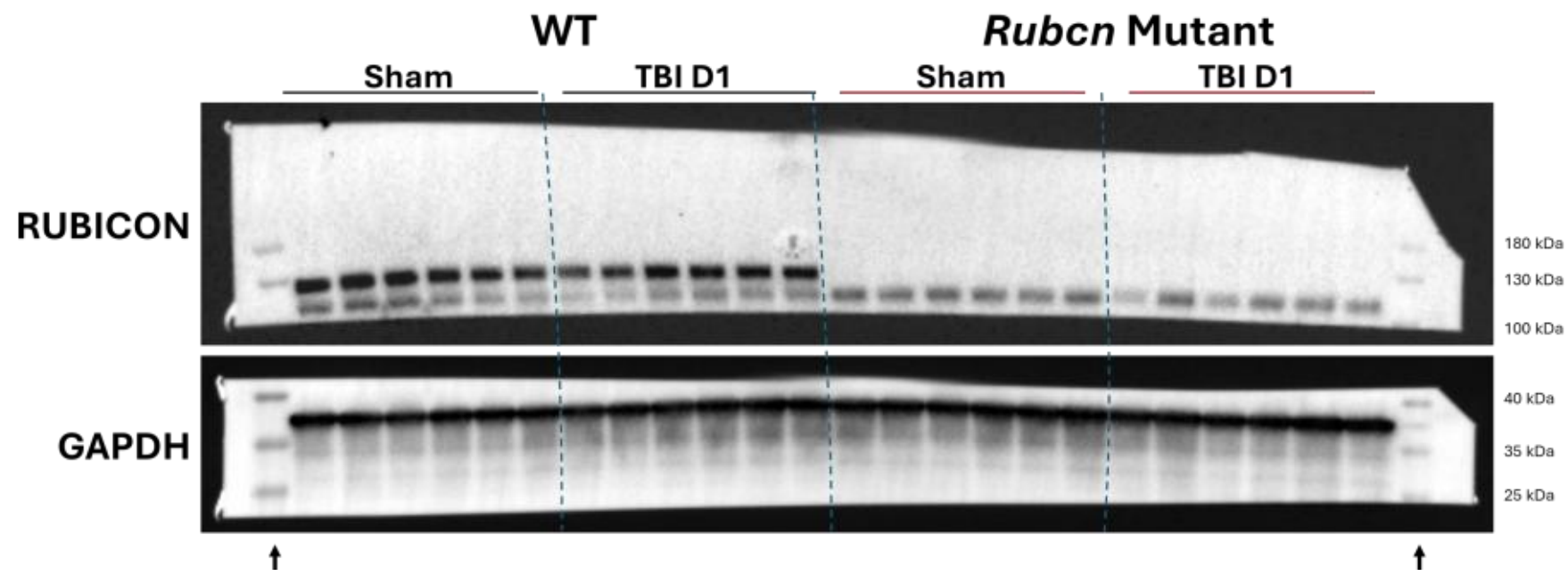
WT
Rubcn Mutant
Sham
TBI D1
Sham
TBI D1
RUBICON
GAPDH
180 kDa
130 kDa
100 kDa
40 kDa
35 kDa
25 kDa

S4B

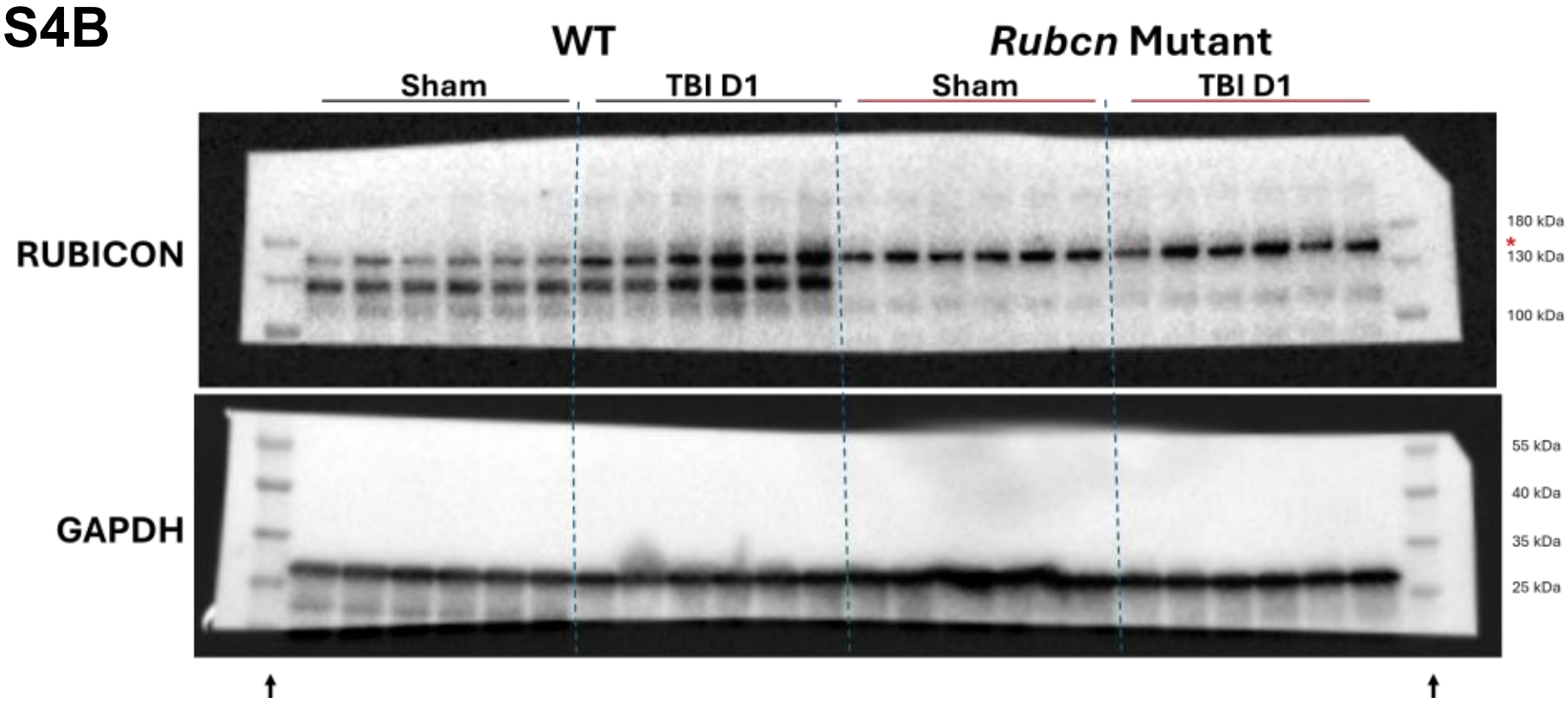
WT
Rubcn Mutant
Sham
TBI D1
Sham
TBI D1
RUBICON
GAPDH
180 kDa
*
130 kDa
100 kDa
55 kDa
40 kDa
35 kDa
25 kDa

S4F (1st replicate)

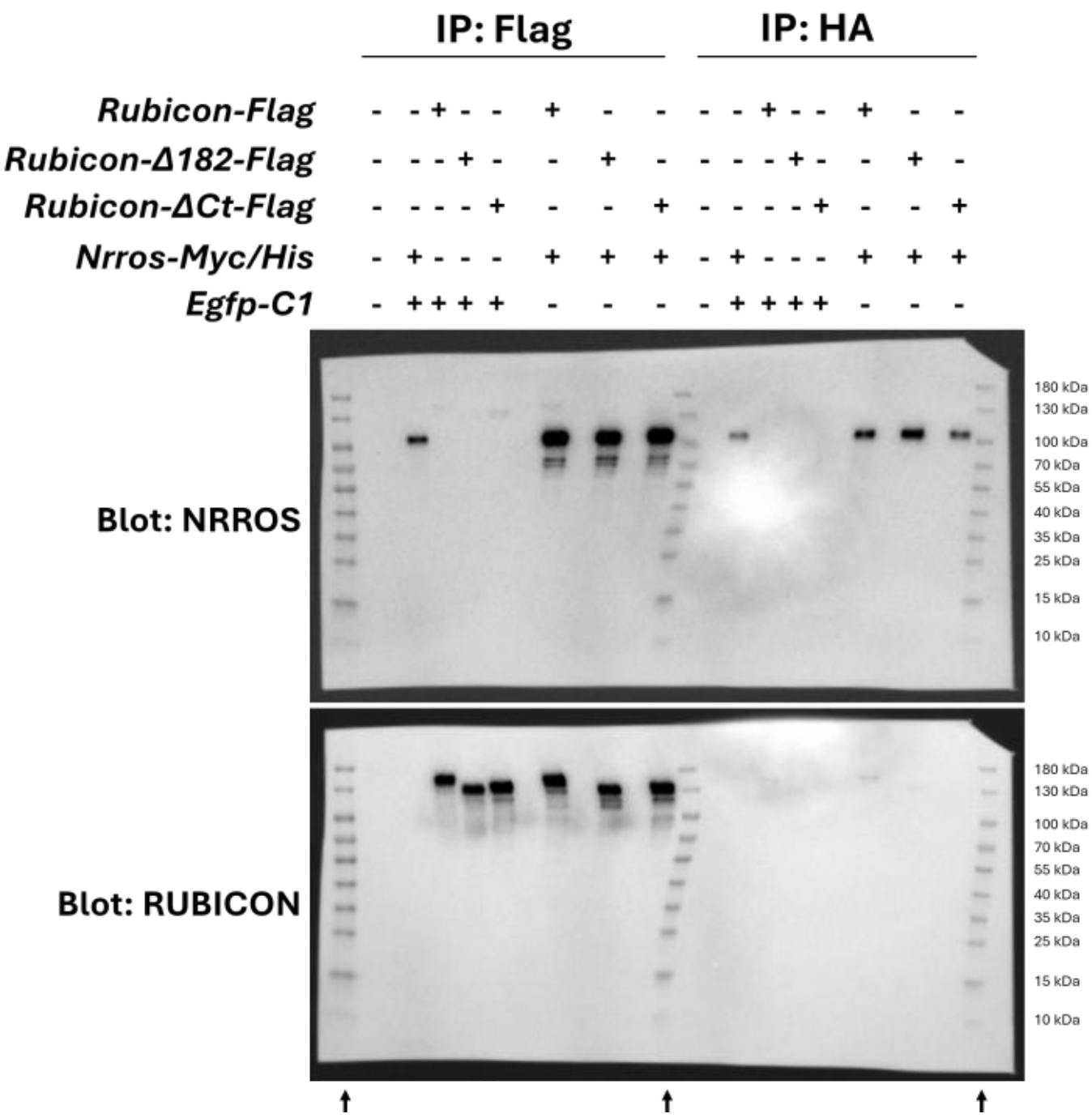

S4H

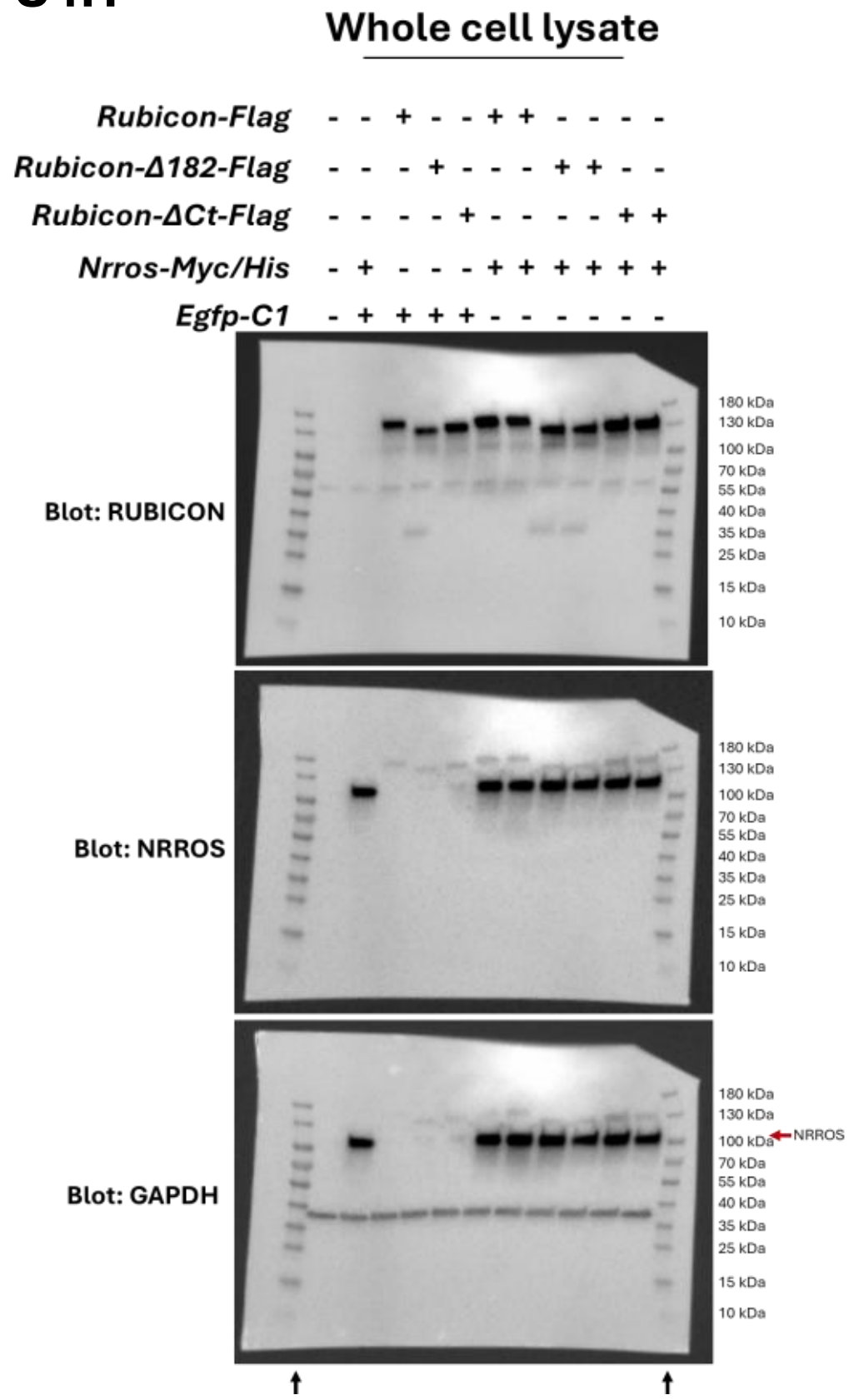

S4F (2nd replicate)

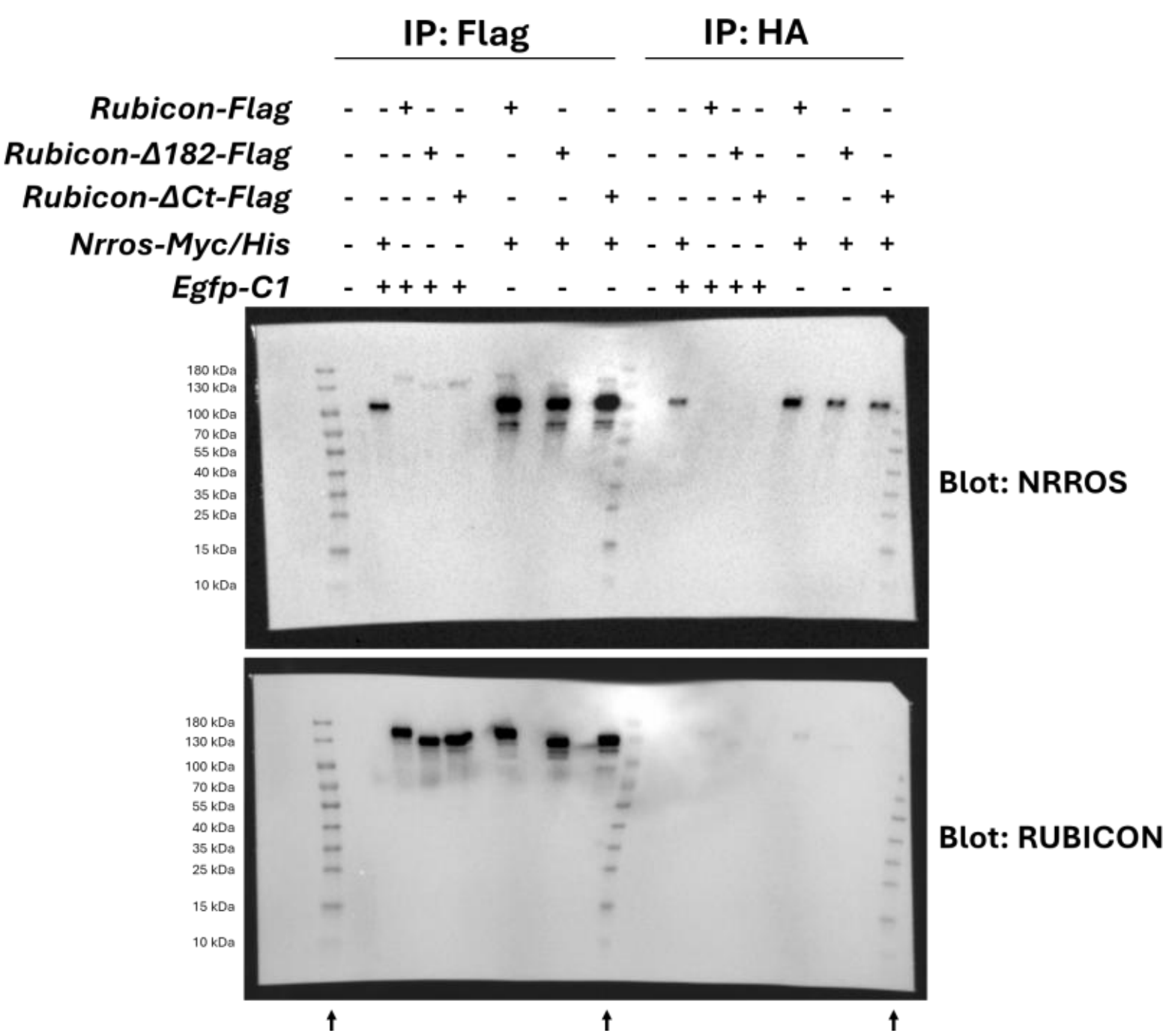

Supplement: Supplementary file 2 [file 41419_2026_9083_MOESM3_ESM.pdf]
